# Sub-1-volt, reconfigurable Gires-Tournois resonators for full-coloured monopixel array

**DOI:** 10.1038/s41377-026-02228-2

**Published:** 2026-02-28

**Authors:** Joo Hwan Ko, Hyo Eun Jeong, Serim Kim, Doeun Kim, Se Yeon Kim, Young Jin Yoo, Hyeon-Ho Jeong, Young Min Song

**Affiliations:** 1 School of Electrical Engineering and Computer Science, Gwangju Institute of Science and Technology, Gwangju, Republic of Korea; 2 Department of Mechanical Engineering, Massachusetts Institute of Technology, Cambridge, MA USA; 3 School of Electrical Engineering, Korea Advanced Institute of Science and Technology, Daejeon, Republic of Korea; 4 GIST InnoCORE AI-Nano Convergence Initiative for Early Detection of Neurodegenerative Diseases, Gwangju Institute of Science and Technology, Gwangju, Republic of Korea; 5 Department of Semiconductor Engineering, Gwangju Institute of Science and Technology, Gwangju, Republic of Korea

**Keywords:** Nanocavities, Nanophotonics and plasmonics, Displays

## Abstract

Achieving vibrant, energy-efficient colour modulation across micrometre-scale pixels is a critical challenge in modern display technology. Conventional approaches face limitations in scalability, high operating voltages, and light loss. Emerging monopixel designs with active materials promise a path to dynamic colour modulation without these drawbacks. However, achieving uniform, energy-efficient colour modulation across the full visible spectrum has remained difficult. Here, we introduce a full-colour, electrically reconfigurable Gires-Tournois (*r*-GT) resonator integrated with the conductive polymer (polyaniline, PANI), representing a significant advance in monopixel display technology. This system enables modulation of complex refractive indices within a sub-1-volt range, producing vibrant colour shifts that exceed complementary hue ranges. The *r*-GT resonator operates at CMOS-compatible voltages with ultralow-power consumption (90 μW cm^−2^), offering scalability from ultrahigh pixel densities (~16,900 PPI) to wafer-scale fabrication. Furthermore, PANI’s metastable states enable memory-in-pixel operation, significantly reducing energy consumption compared to emissive displays. The successful demonstration of a 5 × 5 monopixel array system validates its potential for scalable, energy-efficient, and high-performance photonic applications.

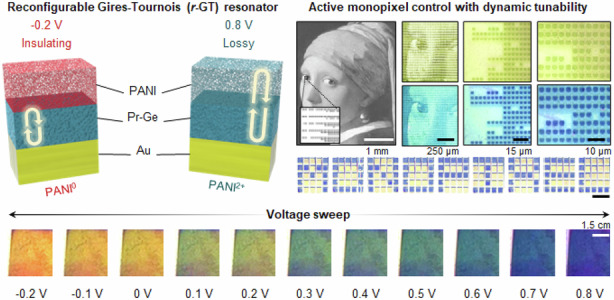

## Abstract (web summary)

Electrically reconfigurable *r*-GT resonator integrated with PANI enable sub-1 V, ultralow-power, full-colour monopixel modulation with individually addressable monopixel array, enabling scalable, memory-in-pixel reflective displays with high pixel density.

## Introduction

From vast electronic billboards to immersive mixed reality eyewear, ensuring consistent colouration and efficiency across pixel scales without degradation remains a key challenge in display technology^1^. It is particularly stringent in near-eye displays, where the need for precise visual reproduction in a few micrometres pixel size is critical, often leading to challenges such as decreased external quantum efficiency and low yields, such as from pick-and-place methods in light-emitting diode (LED) applications^2^. Reflective display technologies, such as liquid crystal on silicon (LCoS), have emerged as promising solutions to these challenges, offering exceptional spatial resolution and precise pixel control. LCoS displays are widely used in commercial products like Google Glass and Magic Leap^3,4^ due to their ability to maintain performance at high pixel densities. However, they typically operate at higher voltages (~6 V) compared to CMOS-compatible standards (~3.3 V), resulting in significant energy losses. Additionally, their reliance on subpixel configurations and thick liquid crystal layers causes light loss and reduces brightness, making further downsizing and scalability difficult due to increased optical leakage and reduced subpixel separation^5^.

Recent advances in ‘monopixel’ reflective displays, which integrate optically active materials into photonic systems, represent a source of active/vibrant colour in the standalone pixel, facilitating flexibility in dimensional scaling^6,7^. For example, the nanopixel array based on phase-change materials (PCMs) like Ge-Sb-Te is designed for ultra-small pixels on the scale of hundreds of nanometres^8^. However, PCM modulation is challenging due to heat transfer dependencies, causing fluctuations in response times that vary with pixel size and limiting addressability and reversibility in electrically operated devices^9^. Meanwhile, electro-optic (EO) materials enable rapid colour changes up to 2.5 MHz and immediate responses under a uniform electric field, independent of pixel size. However, they require an operating voltage above ~30 V for full-colour tuning and face limitations in modulation capacity due to minimal refractive index variations and challenges in light-matter interaction^10,11^. Conductive polymers, such as polyaniline (PANI)^12–14^ or poly (3,4-ethylene-dioxythiophene):polystyrene sulfonate (PEDOT:PSS)^15^, have emerged as promising alternatives, enabling uniform and energy-efficient colour modulation with minimal energy requirements ( <1 V and <0.3 mW cm^−2^)^16^. While they provide remarkable refractive index variation within the visible wavelength range, standalone conductive polymers lack the light-matter interaction required for sufficient colour switching over a broad hue range. Attempts to overcome these limitations using plasmonic or Mie resonators have shown full-coloured great colour variations but are hindered by costly, complex manufacturing processes, hindering manufacturable at actual device sizes. Additionally, various planar resonators have shown potential for colour modulation, though their range remains limited. To enhance interactions with interlayers, the insertion of a lossy layer is employed, which induces significant, non-trivial phase shifts leading to notable colour changes. However, the process is complicated by an intensified oxidation environment during the oxidation-reduction reactions of conductive polymers, highlighting the critical need for passivation.

Here, we introduce an electrically reconfigurable Gires-Tournois (*r*-GT) resonator integrated with PANI, representing a significant step forward in monopixel reflective display technology. The *r*-GT resonator exhibits sharp and strong resonances based on the impedance matching condition, resulting in vibrant colour shifts exceeding a complementary hue change (ΔHue ~220.6°), operating within a CMOS-compatible voltage range (−0.2 V to 0.8 V) and consuming only 90 μW cm^−2^. Furthermore, simulation results show that diverse material combinations can extend the colour gamut to fully encompass the standard RGB (sRGB) space. By stabilizing the *r*-GT resonator interfaces during redox reactions, we address challenges of chromatic degradation and scalability, enabling operation across diverse scales, from micro-pixels (1.5 μm, ~16,900 pixels per inch (PPI)) to centimetre-scale image printing. Furthermore, the metastable states exhibited by PANI contribute to optical memory retention at the pixel level, achieving energy savings up to 7.2 times greater than emissive-type displays. These features, validated through an electrically independent addressing system for a 25-pixel array, underscore the potential of this monopixel *r*-GT resonator for scalable, high-performance photonic applications.

## Results

### Concept of reconfigurable Gires-Tournois monopixels

**Fig. 1:**
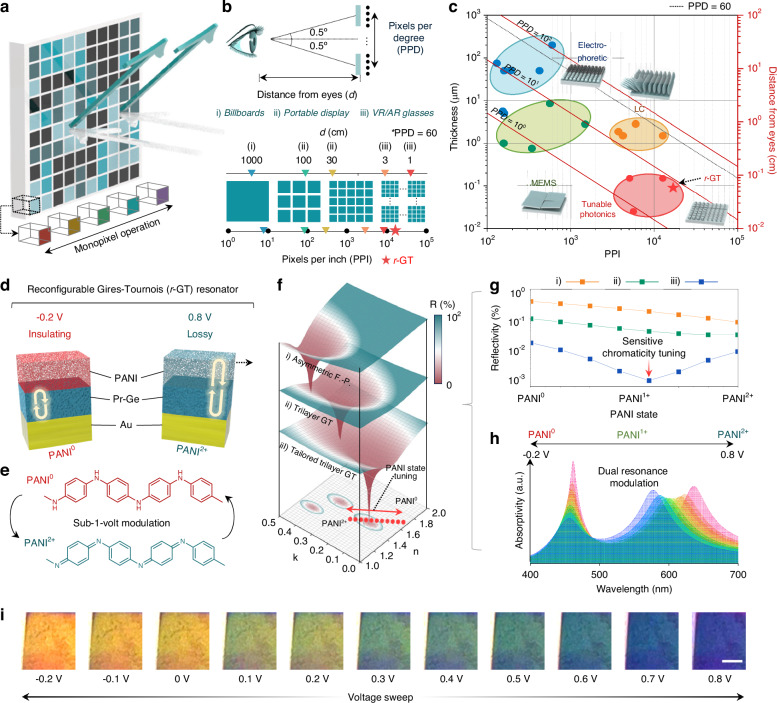
Reconfigurable Gires-Tournois monopixel array. **a** Schematic of the reconfigurable Gires-Tournois (*r*-GT) monopixel array and the reflective light showing full-colour chromaticity. **b** The relationship of pixels per degree (PPD) corresponds to the distance from the eyes (*d*), which determines the minimum pixel pitches (*P*). For example, pixel density ranges required for devices from electronic billboards to VR/AR glasses vary from 87 pixels per inch (PPI) to 10,000 PPI, respectively. **c** Comparison of pixel thickness and its PPI of the electrically addressable pixel array and/or display. The right-side axis represents the distance from the eyes, and the red solid lines indicate the corresponding PPD, ranging from 10^0^ to 10^2^ at each PPI level (See Fig. S1 (Supplementary Information) for details). **d** Schematic of the layered structure of *r*-GT cell consisting of a trilayer resonator (Au/Pr-Ge/PANI). The optical state of PANI determines the resonance mode of *r*-GT. **e** The molecular configuration of PANI and its electrically switchable optical state, alternating between insulating (PANI^0^), intermediate state (PANI^1+^), and metallic states (PANI^2+^). **f** Simulated reflectance of various resonator configurations (Asymmetric F.-P. cavity, Trilayer GT resonator, and Tailored trilayer GT resonator). The scattered dots indicate the complex refractive indices (*n*) and extinction coefficient (*k*) of PANI under different redox states. **g** Reflectivity of *r*-GT in various configurations: ⅰ) asymmetric F.-P. resonator, where PANI is applied directly to an Au substrate; ⅱ) trilayer GT resonator, which includes a dense amorphous Ge layer between Au and PANI; ⅲ) tailored trilayer GT resonator, consisting of a porous Ge layer between Au and PANI. Reflectivity changes as PANI cycles through different redox states from PANI^0^ to PANI^2+^ under resonant wavelength. **h** Absorptivity of *r*-GT corresponding to the change of redox state of PANI. The red curves represent the absorptivity spectra in the fully reduced state of PANI, while the blue curves correspond to the fully oxidized state. **i** Photo images of the colour variation of *r*-GT. Scale bar is 1.5 cm (more experimental results in movie S1 (Supplementary Information))

Reflective displays offer a compact and efficient design, leveraging external light without an internal light-emitting part. It is particularly advantageous for micrometre-scale displays, as it avoids significant challenges associated with optical efficiency degradation and fabrication yield, especially in pixel dimensions down to a few micrometres. Moreover, advancements in structural colour-based reconfigurable photonics facilitate monopixel functionality, obviating the necessity for subpixel arrangements and enabling the fabrication of extremely small pixel dimensions (Fig. 1a). Considering the required PPI for adequate image quality, which varies with the distance (*d*) between the display and the observer’s eye based on general guidelines, a pixels per degree (PPD) of at least 60, is typically necessary to ensure continuous and high-quality display output based on 20/20 vision standard^17^. Additionally, recent studies suggest that achieving maximum resolution for near-eye displays, such as those used in virtual and augmented reality (VR/AR), may require a significantly higher PPD of over 90, necessitating pixel sizes as small as 2 μm, corresponding to ~10,000 PPI (Fig. 1b)^18^. As illustrated in Fig. 1c, this trend aligns with the structural characteristics of the types of reflective displays, e.g., electrophoretic display^19–22^, electrowetting display^23–25^, micro-electro-mechanical system (MEMS)^26–29^, liquid crystal (LC) display^30–33^, where the minimum feasible pixel size typically inversely correlates with the required thickness of the device, considering issues like optical leakage and misalign issues (Supplementary Information Fig. S1). Moreover, as the baseline PPD increases, the PPI required for a distance (*d*) tends to increase proportionally, suggesting that the closer the display is to the eye, the exponentially greater the required PPI becomes. In this context, the ability of the *r*-GT resonator to achieve high PPI dimensions (16,900 PPI), particularly as it has been implemented without structural or optical degradation, is notable.

The outstanding abilities, including downscaling ability and vibrant colouration of the *r*-GT resonator, originate from three major points: (i) the ultrathin scale of the *r*-GT, (ii) the strong coupling between the conductive polymer and the GT resonator, and (iii) the sensitive shift in the resonance wavelength. The *r*-GT resonator developed in this study consists of a trilayer ITO/PANI/Pr-Ge/Au architecture, where the engineered Pr-Ge lossy layer enables near-ideal impedance matching and supports a sharp resonance that responds sensitively to the redox-induced optical changes of PANI (Supplementary Information Fig. S2). The GT resonator, which has been recently studied as a flat metasurface for generating strong resonance by controlling the speed of light, is utilized as a core photonic structure to integrate active material (PANI) strongly, resulting in a sensitive adjusting ability of resonances from single to dual mode (Fig. 1d)^34,35^. During voltage sweeps in cyclic voltammetry, PANI undergoes two reduction/oxidation steps, displaying three redox states: PANI^0^ at −0.2 V, PANI^1+^ at 0.4 V, and PANI^2+^ at 0.8 V, including continuous complex refractive index variation (Fig. 1e). PANI’s significant refractive index change (∆*n* ~ 0.3, ∆*k* ~ 0.6) during these transitions addresses the limitations of standalone conductive polymers, which do not provide sufficient light-matter interaction for effective full-colour switching across a broad hue range (Supplementary Information Fig. S3). Thus, the GT resonator, integrated with PANI, is engineered to enable sensitive resonance shifts for enhanced colour purity in the additive colour regime, and to produce a precise and strong resonance with requiring only a 90 nm thickness of PANI, achieving extremely low power consumption (<90 μW cm^−2^, Supplementary Information Figs. S4 and S5)^36^. For example, as illustrated in Fig. 1f, various GT resonator configurations are compared to a conventional Fabry–Perot cavity. The conventional GT resonator exhibits a single resonance in the visible spectrum with limited sensitivity to absorptivity modulation, as shown in the bottom contour plot and Fig. 1g. In contrast, the trilayered GT resonator, which incorporates a lossy interlayer to better match the optical impedance between interfaces, demonstrates enhanced resonance intensity and supports dual resonances within the visible range. However, its resonance peaks are relatively broad, limiting its ability to achieve high colour purity. On the other hand, the tailored trilayered GT resonator incorporating a porous Ge layer exhibits nearly perfect impedance matching, leading to near-unity absorption. This configuration enables strong resonance tuning in response to the redox activity of PANI, resulting in significantly higher colour purity due to the formation of narrow and well-defined spectral peaks (Supplementary Information Figs. S6–8). Furthermore, Numerical simulations confirmed that the device maintains consistent colour performance over a broad range of incident angles, corresponding to an effective field of view (FOV) of ~60° (−30° to 30°), demonstrating its potential applicability to commercial reflective displays, such as Magic Leap, which features an FOV of ~50° (Supplementary Information Fig. S9)^37^.

By adjusting the redox states of PANI, one can finely tune the resonance conditions in the *r*-GT, achieving extensive hue modulation (Fig. 1h and Supplementary Information Fig. S10). Figure 1i highlights its colour dynamics corresponding to the voltage range even below 1 V (Movie S1, Supplementary Information Figs. S11 and S12). Figures S13 and S14 (Supplementary Information) show a tendency of a gradually adjusted resonance mode corresponding to the modulation in PANI’s state. Considering that a newspaper typically exhibits a reflectance contrast of ~40%, our device demonstrates practical performance with ~40% reflectance in the OFF state and ~80% in the ON state^38,39^. The detailed design process will be discussed in the next section.

Building on these capabilities, the *r*-GT resonator stands out by achieving significant chromatic variation of 220.6° at sub-1-volt levels. It exceeds the complementary colour range, which is essential for enhancing the perception of depth and realism in digital art and displays^40^. This performance is notable compared to previous technologies, including active photonics integrated with redox-based tunable materials (e.g., conductive polymer), phase change/transition materials, reconfigurable photonics via dimension tuning (e.g., electrically responsive photonic crystals), and electrostatic inkjet control (e.g., electrophoretic displays), highlighting its advanced capability in precise colour modulation across a wide spectrum with dimensional downscaling into micrometer-scale (Supplementary Information Tables S1 and S2) Furthermore, the thin-film *r*-GT resonator exhibits a wide colour modulation efficiency per unit power compared to nanostructured photonic structures such as metasurfaces and nanocaves (Supplementary Information Fig. S15, Tables S3 and S4)^8,9,41,42^.

### Full-colour expression of *r*-GT

**Fig. 2:**
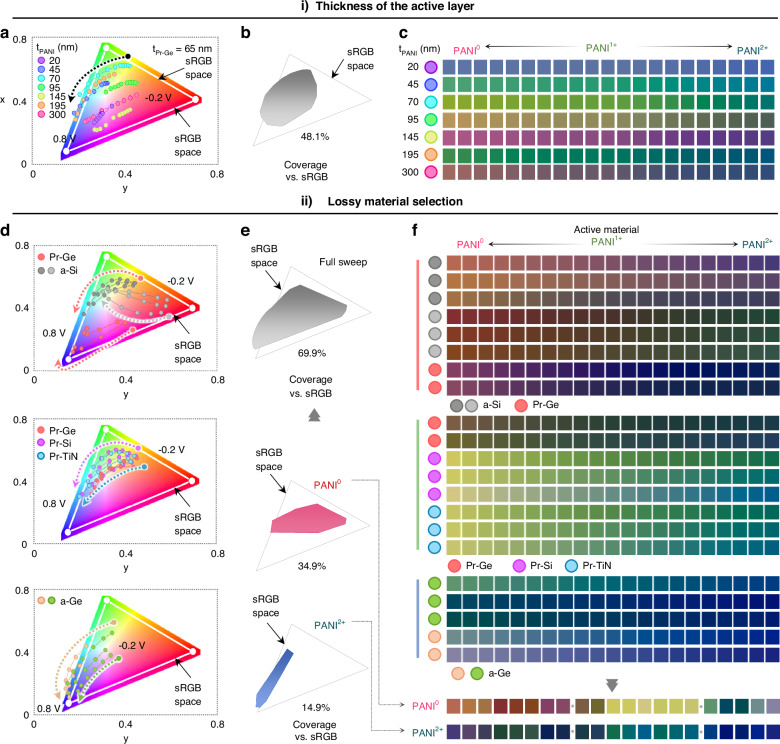
Full-colour operation of *r*-GT. **a** CIE plot of the *r*-GT with lossy layer (Pr-Ge). The white triangle represents the standard RGB (sRGB) colour space, and the dashed line shows the voltage-dependent shift of the colour coordinates from −0.2 V to 0.8 V. **b** sRGB colour gamut coverage of *r*-GT across different states of PANI. **c** Colour palette associated with each configuration. **d** CIE chromaticity plots for various configurations of the *r*-GT with different material combinations. **e**, **f** sRGB colour gamut coverage (**e**) and associated colour palettes (**f**) of *r*-GT devices tailored to represent primary red, green, and blue (R, G, B) colour via PANI redox modulation

This section delves into the vibrant colour modulation abilities and the universality facilitated by diverse structural combinations within the *r*-GT. The pronounced chromaticity changes in the *r*-GT are attributable to sensitive adjustments in resonance conditions, driven by each layer’s precise engineering based on transmission line theory, allowing exact monitoring of the interactions between light and the layered structures. Notably, the introduction of a porous (Pr)-Ge structure in the lossy material medium enhances the responsiveness of the modulation environment to changes in PANI’s complex refractive index (Supplementary Information Note 1; Figs. S16 and S17). As a result, the *r*-GT’s reflectance shows a marked negative group delay, effectively preventing chromaticity cancellation in the absorptive state (Supplementary Information Note 2; Fig. S18). This mechanism contributes to strong and consistent colour modulation. Figure 2a, b display chromaticity covering as thickness of PANI and redox state transitions through from PANI^0^ to PANI^2+^, illustrating 48.1% of sRGB coverage during these transitions (Supplementary Information Figs. S19 and S20). The colour palette visually represents the range of colour modulation achievable within a single structure, highlighting the importance of active material thickness and selecting an optimal thickness (Fig. 2c). Moreover, various material combinations have been utilized to enhance colour expression, achieving performance that spans the sRGB colour space. Each colour dot in the figure represents a configuration involving different combinations of lossy materials within the *r*-GT, highlighted by the white triangular line that marks the sRGB range (Supplementary Information Fig. S21). Figure 2d, e quantify the sRGB colour gamut coverage of the *r*-GT across PANI’s various states, noting that the full sweep of redox states covers 69.9% of sRGB, enabling vibrant colour expression. Finally, Fig. 2f visually presents the colour palette for each configuration, demonstrating the substantial variation and impact of these material integrations, including the primary RGB colours.

### Self-passivation layer with enhanced stability and fast response time

**Fig. 3:**
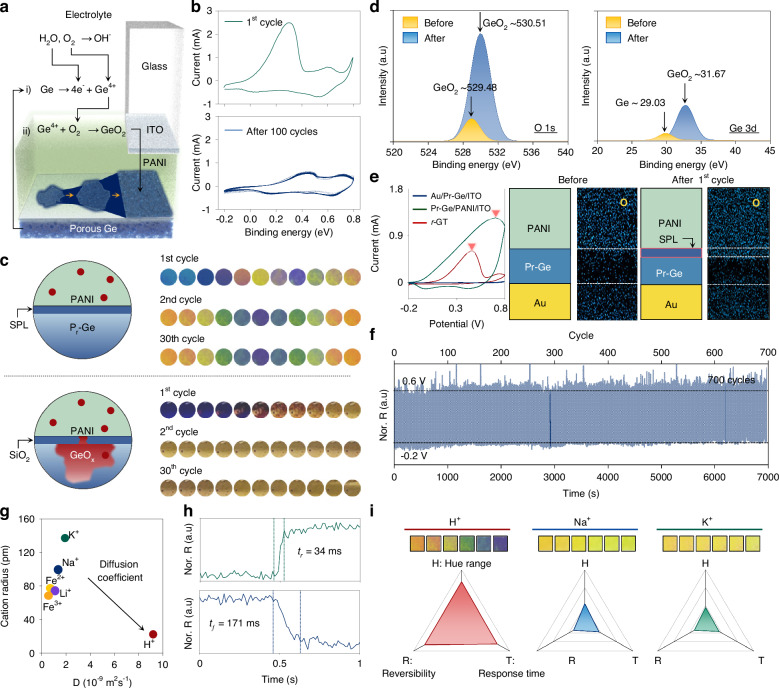
Characterization of the self-passivation layer (SPL) of *r*-GT. **a** Illustration of the formation of SPL. The porous Ge layer undergoes oxidation through electrons and oxygen. **b** Cyclic voltammetry (CV) curve of the *r*-GT at first cycle (red line) and after 30 cycles (blue line) with a reference electrode (RE, Ag/AgCl). A strong oxidation peak occurs during the initial oxidation stage, followed by a stable electrochemical response in subsequent cycles. **c** Schematic of the permeability of photons through the SPL and SiO_2_ layer, along with corresponding photo images of the reflective colours. **d** XPS spectra of O 1 s and Ge 3 d at the interface between PANI and porous Ge layer. The blue line represents the state of the *r*-GT before the redox reaction, while the red line shows the state after the reaction. **e** CV curve of Au/Pr-Ge/ITO (blue line), Pr-Ge/PANI/ITO (green line), and *r*-GT resonator (red line) at a scan rate of 0.05 V s^−1^. Energy dispersive spectroscopy (EDS) analysis of a cross-sectional *r*-GT. The EDS mapping reveals that the atomic percentage of oxygen (blue) is increased in the SPL region after the redox reaction. **f** Reflectance spectrum with potential cycled from −0.2 V to 0.6 V (interval: 10 s) over 700 cycles. **g** Comparison of various cations with photon based on cation radius and diffusion coefficient. **h** The electrically responsive reflectance exhibits a switching speed with a rise time of 34 ms and a fall time of 171 ms, respectively. **i** Comparison of different cation-based electrolytes in terms of hue range (H), response time (T), and reversibility (R)

Protons are essential for doping PANI, enabling fast diffusion and enhanced modulation speed; however, they also create an acidic environment, which may result in structural corrosion^43^. To protect against the corrosion issue, Fig. 3a illustrates the irreversible, intentionally applied oxidation of the Ge layer during the first cycle of voltage sweeps in cyclic voltammetry. The oxygen ions and electrons involved in oxidation are derived from the electrolyte and the conductive substrate, resulting in the formation of the GeO_2_ layer through processes (i) and (ii)^44^. As shown in Fig. 3b, the electrochemical reactions of the *r*-GT monopixel are examined during 100 cycles. The cyclic voltammetry curves show a high current peak intensity at a specific voltage during the first cycle, followed by stable redox reactions in the subsequent cycles (Supplementary Information Fig. S22). This phenomenon suggests that partial oxidation of Ge occurs, leading to the formation of the self-passivation layer (SPL) to protect its porous structure from acidic electrolyte^45^. During the first cycle of the cyclic voltammetry reaction, the peak current density of the *r*-GT resonator at 0.45 V points to the oxidation of Ge to GeO_2_, in response to oxygen sources being absorbed during the deposition process and/or hydroxyl groups diffusing from the electrolyte^46,47^. To confirm the necessity of the intentionally formed SPL through Ge oxidation, the device is fabricated using the conventional material (SiO_2_) for comparison. Under acidic conditions, the Ge medium is fully oxidized, resulting in degraded electrochromic properties. On the other hand, SPL-based *r*-GT provides great protection ability, showing great reversibly switchable ability, enabled by GeO_2_ passivation with inherently lower interface defect density than SiO_2_ passivation^48–50^. (Fig. 3c). As proof of our suggestion, energy dispersive spectroscopy (EDS) and X-ray photoelectron spectroscopy (XPS) were conducted, revealing a corresponding increase in the GeO_2_ proportion in the XPS O 1 s signal (Supplementary Information Fig. S23). In addition, the Ge 3 d photoelectron spectrum before (blue line) and after redox reaction (red line) show a change in spectral intensity with a shift from the initial state to a higher binding energy corresponding to the GeO_2_ peak profile (Fig. 3d). As shown in Fig. 3e, comparison of the CV curves for Au/Pr-Ge/ITO, Pr-Ge/PANI/ITO, and the *r*-GT resonator reveals redox peaks in the PANI-containing structure relative to structure without PANI. In addition, the structure containing Au effectively suppresses excessive oxidation of Pr-Ge, enabling the formation of SPL. These results closely match the EDS mapping profile in terms of oxygen and nitrogen atomic proportion. Figure S24 (Supplementary Information) also presents EDS results showing an increase in nitrogen content, which occurred simultaneously with the doping/de-doping process. Figure 3f shows that the reflectance spectrum repeatedly activates and reverts to its initial state and different voltages (−0.2 V and 0.6 V, interval is 10 s) during 700 cycles (Supplementary Information Figs. S25 and S26). Figure 3g shows that proton has the smallest cation radius and largest diffusion coefficient in comparison with other cations, resulting in a fast response time of doped in just 34 ms (oxidation) and effectively de-doped to the reduced state within 171 ms (reduction) (Fig. 3h; Supplementary Information Figs. S27 and S28). The *r*-GT resonator is measured into the other electrolyte containing either sodium ion (Na^+^) or potassium ion (K^+^), which have larger ionic radii and lower diffusion coefficients compared to protons. Figure 3i displays a radar chart showing that both Na^+^ and K⁺-based electrolytes exhibit overall lower performance in terms of modulated hue range (H), response time (T), and reversibility (R). Building on the results, it suggests that proton-based electrochemical reactions guarantee sufficient redox responses with fast reaction speeds. Although they may be limited by corrosive properties, we have overcome this issue using SPL, thereby expanding the range of applicability for PANI-based photonics (Supplementary Information Table S5; Figs. S29 and S30).

### Flexibility in scaling the dimensions of the r-GT resonator

**Fig. 4:**
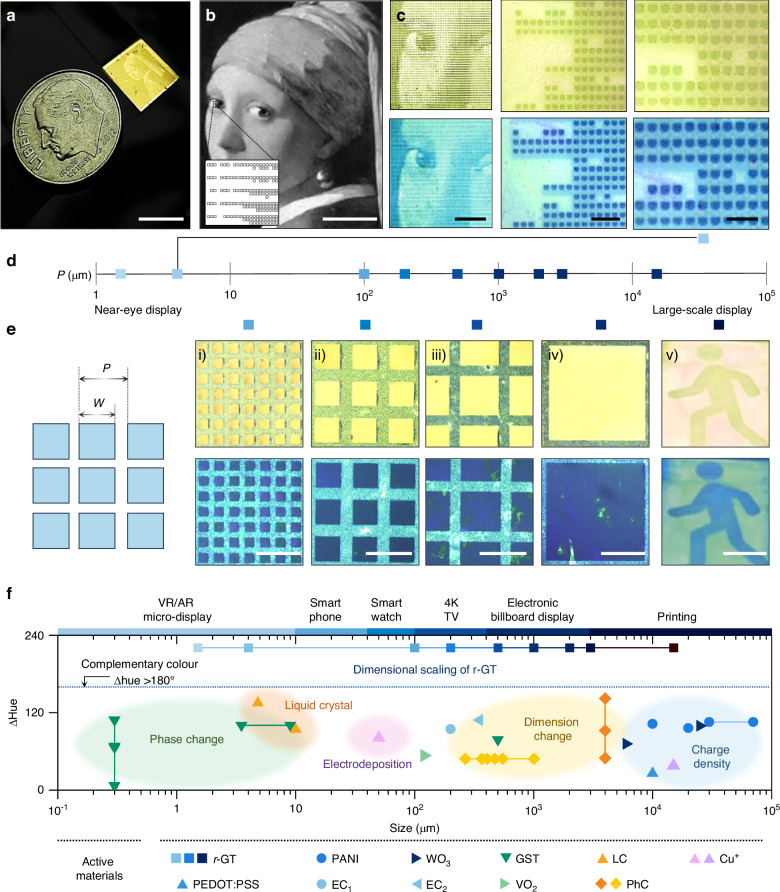
Electrically triggered optical response and programmable memory functions. **a** Photograph of the high-density (HD) patterned *r*-GT resonator alongside a 1-dime coin. **b** Bayer-pattern image of the painting “Girl with a Pearl Earring” with a 16-level scale. **c** Microscope image of a selected region at a voltage of HD *r*-GT array. The bluish image shows the *r*-GT in the PANI^2+^ state. **d** Recommended scale diagram of the pixel size corresponding to commercial display devices. **e** Optical microscopy images of each pixel showing electrically switchable dual states and the electrochromic emergency exit sign. **f** Comparison of optical modulation range versus span (~220.67°) of the operated pixel size with various tunable photonics systems. Scale bars, **a** 1 cm, **b** 1 mm, **c** 250 μm, 15 μm, and 10 μm. **e** ⅰ–ⅳ 300 μm and ⅴ 5 mm

The optical/chemical robustness of *r*-GT offers the flexibility in scaling the pixel dimension from centimeter-scale to micrometer-scale. Particularly, in this section, we demonstrate the diverse scale of the reconfigurable pixel array with a colour switching function. Figure 4a shows the Bayer-pattern with 16-unit dots, incorporating the painting *Girl with a Pearl Earring* (Johannes Vermeer), achieving a compact panel size smaller than a 1-dime coin. By varying the density of unit dots, the spatial fill factor can be adjusted to represent 16 graded intensity levels (4-bit) in the corresponding pixel array (Fig. 4b; Supplementary Information Fig. S31a–d). The 5 µm square pattern is defined as a unit pixel, and the array patterns display a clear high-density (HD) image (Fig. 4c; Supplementary Information Fig. S32). The chromaticity of the HD patterned *r*-GT resonator can be adjusted from yellow to blue under sub-1-volt conditions (Supplementary Information Fig. S31e). Quantitatively, the pixel size can be classified according to target applications by considering the distance between the display and the observer’s eyes. In this regard, we experimentally confirmed diverse sizes of pixels with the colour switching functionality. Figure 4d, e illustrates the pixel size distribution ranging from the few micrometer-scale required for VR/AR displays to the centimeter-scale needed for electronic billboards^51,52^. The active area of *r*-GT is successfully patterned with different pixel sizes: i) *P* = 160 µm; ii) *P* = 370 µm; iii) *P* = 630 µm; iv) *P* = 1300 µm; and v) centimeter-scale image printing. Additionally, the experimental validation of scaling down has been confirmed up to a pixel pitch of 1.5 µm, achieving 16,900 PPI (Supplementary Information Figs. S33 and S34). Diverse sizes of each pixel are compared with previously reported reflective-type displays with pixel operation based on reconfigurable photonics using various types of active materials, such as electrochemical doping material (PANI^53–55^, PEODT:PSS^56^, WO_3_^57,58^), phase-change materials (GST^8,9,59^, VO_2_^60^), liquid crystal (LC)^61,62^, dielectric (SiO_2_) nanoparticles-based photonic crystal^63,64^, and metal electrodeposition^65,66^ (Fig. 4f; Supplementary Information Fig. S35**;** Table S6). As described, depending on the type of active materials, the feasible dimensions can be influenced. For example, when adjusting the period of a photonic crystal based on dielectric nanoparticles (i.e., categorized as a dimension change), there is a required vertical dimension, which may limit the adjustments in the lateral dimension. On the other hand, examples based on PCMs, which can alter colour by applying localized heat, have experimentally demonstrated very small pixelization down to ~300 nm. However, in this case, the heating is applied via an atomic force microscope tip, and there is a lack of circuit-based pixel-by-pixel addressing capability. Also, from the viewpoint of colour adjusting, despite vibrant colour changes, there are no cases that achieve a sufficient complementary range (ΔHue > 180°). This may be due to ⅰ) the limitations in the change in optical constants of the active materials themselves, or ⅱ) the absence of synergistic coupling with the photonic structure necessary to induce sufficient light-matter interaction. In this context, *r*-GT successfully demonstrates great flexibility in scaling profits for diverse types of applications and also provides an extensive colour modulation.

### Addressable characteristics of *r*-GT monopixel array

**Fig. 5:**
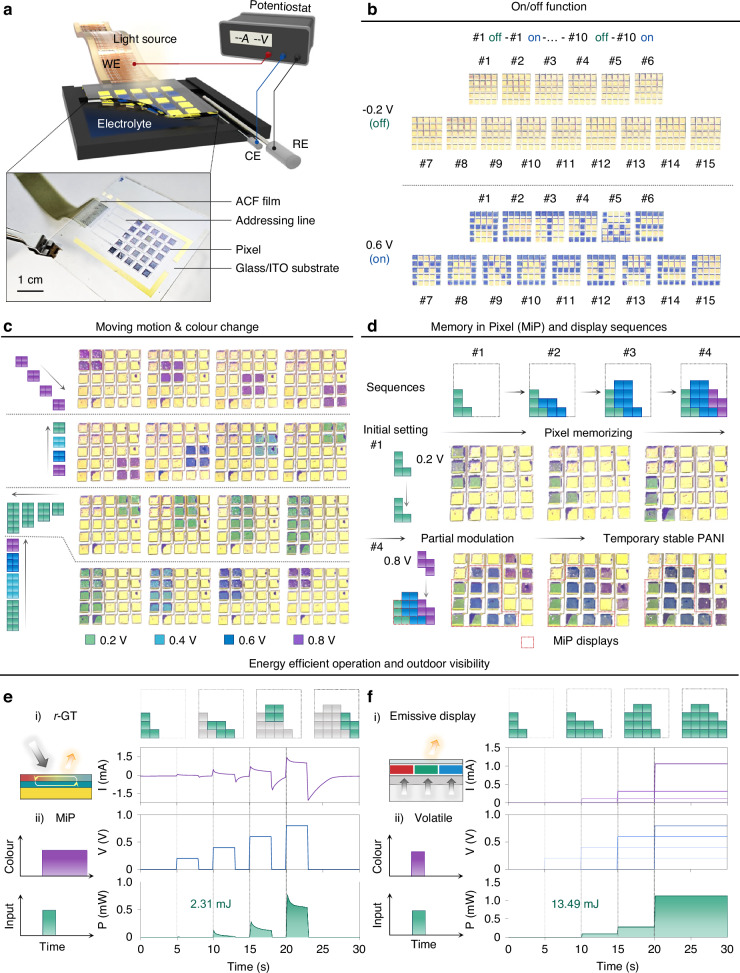
Electrochemical characterization and colouration performance of the *r*-GT monopixel array. **a** Schematic illustration of the optical measurement setup for the *r*-GT resonator and the photo images of a 5 × 5 *r*-GT resonator array. **b** Photograph of the individually controlled pixel array with on/off function, which represents letters of “ACTIVE MONOPIXEL”. **c** Photo images of electrically addressable pixel array with pre-programmed scenarios including moving motion and colouration. **d** Optical memory properties of the *r*-GT monopixel for sequential visualization of the famous game of Tetris tasks over time. **e**, **f** Comparison of the power consumption of the *r*-GT resonator (**e**) and that of commercial light-emitting diode (LED) during sequence processing (**f**)

To demonstrate the addressable *r*-GT array, we fabricated a 5 by 5 pixel array, each pixel measuring 9 mm². Figure 5a presents a schematic and a photographic image of the *r*-GT array, which includes a working electrode (WE), counter electrode (CE), and reference electrode (RE). The potential applied to the WE is transferred via an anisotropic conductive film (ACF) attached to the addressing line (Ti/Au). The CE and RE utilize Pt and Ag/AgCl electrodes, respectively. Figure S36 (Supplementary Information) details the design and mask patterns of the addressable *r*-GT monopixel array. Each pixel is controlled by applying pre-programmed potentials that determine the colours, i.e., −0.2 V for yellow and 0.6 V for blue. By selectively applying the target voltage, the letter pattern ‘ACTIVE MONOPIXEL’ is displayed (Fig. 5b; Supplementary Information Figs. S37 and S38). Figure 5c shows selectively controlled patterns in diverse colours achieved by applying different potentials: 0.2 V for green, 0.4 V for cyan, and 0.8 V for magenta. By applying a series of pre-programmed, selective potentials to pixels with varying potentials and addresses, sequential image patterns are demonstrated. Intriguingly, PANI provides a temporal memory function with a metastable ability that maintains its redox states with open-circuit memory properties for over 4 hours (Supplementary Information Fig. S39)^16^. Leveraging this property, we showcased a Memory-in-Pixel capability^67^, resulting in significantly reduced power consumption for maintaining pre-patterned images. Figure 5d illustrates four series of sequences by demonstrating the game of Tetris being played. Under these conditions, the power density required for colour switching is measured at only 2.31 mJ, eliminating the need for continuous encoding of colour information (Fig. 5e). To compare the power density of the *r*-GT resonator with that of a commercial display, we assumed the power consumption for an LED, calculated at 13.49 mJ, to align with the minimum and maximum power values provided by the *r*-GT resonator (Fig. 5f; Supplementary Information Fig. S40).

As a practical demonstration of reflective display, Fig. S41 (Supplementary Information) illustrates an energy-efficient display with excellent visibility, even in outdoor environments. The figure demonstrates how the *r*-GT resonator, utilizing ambient light as a light source, maintains clear visibility across various lighting conditions, with an illumination range of 5–200 W m^−2^. The captured pictures were converted to grayscale, and intensities were extracted from selected pixels (dashed red line in Supplementary Information Fig. S41d) for highlighting the contrast between letters and the background. In the case of the *r*-GT display, increasing ambient illumination resulted in enhanced contrast, improving visual legibility. In contrast, the LED display exhibited an insufficient contrast ratio to meet the level 5 requirement of the WCAG 2.1 guidelines for optimal visibility in web conditions, as referred to the W3C Web Content Accessibility Guidelines^68^. Consequently, LED displays exhibit lower visibility than this value under high illumination, even at the highest panel brightness with consuming higher power density.

## Discussion

In summary, this work presents a highly efficient monopixel reflective display demonstrating a broad hue range under sub-1-volt level. The structural stability of *r*-GT provides the feasibility of scaling down to micrometer-pixel demonstration (~16,900 PPI), an efficient way for HD near-eye displays^69^. Furthermore, the experimental prototype of addressable array displays has proven its practicality as a highly refined display technology. Also, the metastability further enhances power efficiency, suggesting utility from temporary to long-term image viewing aspects. Inherently, the *r*-GT is a reflective display that utilizes external light sources, significantly enhancing visibility outdoors. The incorporation of a loss layer facilitates improved optical interaction across the tailored trilayer structure, resulting in pronounced colourimetric shifts (Supplementary Information Fig. S42). Merging this feature with its scalability to centimeter levels could extend its potential applications to outdoor electronic billboards (Supplementary Information Fig. S43). Furthermore, integration with emissive materials such as quantum dots^70,71^ or organic LEDs^72^ enables hybrid display architectures^73,74^ that combine the high brightness and colour fidelity of emissive displays with the ultralow-power electrochromic functionality of reflective systems, offering versatile opportunities for multifunctional applications.

The suggested *r*-GTs and their future engineering directions exhibit remarkable applicability; however, certain challenges must be addressed for realistic applications. A notable concern is the scalability to commercial-level devices, potentially up to 4 K or 8 K resolutions, which involve a vast number of pixels and their efficient operation. As the array size increases, the rising number of addressing pixels leads to significant power dissipation, especially in 5 × 5 monopixel array. Incorporating transistors within the pixel structure can enhance selectivity and reduce power wastage, particularly in high-density devices^60^. As the next step, we are integrating *r*-GT resonators into an active-matrix architecture to establish a stable and reliable electrochemical monopixel platform that remains robust even in high-density arrays. Additionally, the requirement for an electrolyte in electrochemical switching introduces vulnerabilities to oxygen and humidity. Modifications to the fabrication process and sealing electrochemical cells in inert gas environments or using solid electrolytes could significantly improve switching performance^75–77^. Such advancements will accelerate the development of more reliable and safe electrochemically active photonic cells^78,79^, moving us closer to their integration in end-to-end commercial product packaging processes.

## Materials and methods

### Optical calculation

The optical simulation for the reflectance and absorption profiles in the *r*-GT resonator was calculated using commercial software (DiffractMOD, RSoft Design Group, USA) employing the rigorous coupled wave analysis method. Material dispersion and complex refractive indices were also considered. The effective refractive indices of PANI and Ge were measured by an ellipsometer (Elli-SEU, Ellipsotech, South Korea) from the electrodeposited thin PANI layer on an Au substrate. The refractive index of Au was obtained from literature^80^. The effective complex refractive indices of the porous Ge layer were calculated using commercial MATLAB software (MathWorks, Inc.) based on volumetric average theory (VAT)^81^.

### Electrochemical setup and measurement

The electrochemical setup was composed of a potentiostat (PARSTAT4000A, AMETEK, USA), counter electrode (Pt mesh and wire), reference electrode (silver/silver-chloride, Ag/AgCl), and electrolyte (0.5 mol L^−1^ NaCl dissolved in 10 mmol L^−1^ HCl). The reflectance spectra and the time-dependent property were measured in 1 M HCl and 2 M NaCl. The setup includes an Xe-lamp (SLS200, Thorlabs, USA) and a spectrometer (OCEAN-HDX-VIS-NIR, Ocean Insight, USA), connected via an optical fiber to collimate the light beam. In addition, electrochemical doping and de-doping processes were performed in situ during the measurements using a potentiostat (PalmSens4, PalmSens, Netherlands).

### Fabrication of *r*-GT resonator

The thin-film PANI layer was deposited in the following steps. Initially, the ITO glass (Omniscience, Korea) substrate was attached with Cu tape to serve as a conductive platform during the electrochemical deposition process. The PANI layer was electrochemically deposited using a potentiostat (PARSTAT4000A, AMETEK, USA) by sweeping the potential from −0.2 V to 0.8 V (scan rate: 0.05 V s^−1^). The electrolyte solution used to grow the PANI layer consisted of 2 M HNO_3_ and 50 mM aniline monomer. During the deposition, the current response against the counter electrode (Pt mesh) was recorded for applied potential versus the reference electrode (Ag/AgCl) (Supplementary Information Fig. S43). Photoresist (AZ 9260, AZ Electronic Materials, Luxembourg) defined the pixel area on the PANI layer through photolithography. The patterned sample was processed with dry etching using a reactive ion etching device (RIE, PLASMA LAB80, Oxford, USA) with CF_4_ gas. To electrically isolate each pixel, the ITO substrate was etched with a wet etchant (CE-100, Transene, USA). After removing the photoresist layer using acetone, the patterned sample was covered with the shadow mask for deposition of the porous layer. To form a porous Ge layer, the glancing angle deposition method was utilized by electron-beam evaporation (KVE-E2000, Korea Vacuum Tech Co., Korea) with a designed tilted sample holder at a deposition angle of 80°. The Au reflector was deposited through another-sized shadow mask. For the addressing *r*-GT monopixel array, the photoresist (AZ 5214, AZ Electronic Materials, Luxembourg) was used with an image reversal process with a pre-patterned Cr photomask. The Au electrode was vertically deposited on the Pr-Ge layer using electron-beam evaporation under a high vacuum (~10^−6 ^Torr). Finally, the electrodes were defined by a lift-off process in acetone (Supplementary Information Fig. S44). To manufacture the HD display, the micropixel pattern was defined on the PANI layer using an image reversal process with the mask aligner (MJB3 UV400, Karl Suss, Germany). The Cr photomask featuring the painting “Girl with a Pearl Earring” was pre-fabricated. The porous layer was deposited to the target thickness with an approximate deposition rate of 1 Å s^−1^ via electron-beam evaporator, followed by a lift-off process to define the patterned structures. Subsequently, Au was deposited across the entire substrate to the desired thickness using a magnetron radio-frequency sputtering system (DDHT-LSH2, Daedong High Tech., Korea) under a high vacuum (~10^−6 ^Torr), covering the entire patterned region. This process simultaneously defined the metallic reflector while providing interfacial area to facilitate ion exchange through the electrolyte.

Electron-beam lithography was performed to define the micropixel array on a positive-tone electron-beam resist (950 PMMA A6, Kayaku Advanced Materials, United States) using a Raith ELPHY Quantum system (Raith ELPHY Quantum, Raith GmbH, Germany) integrated with a scanning electron microscope (SU5000, Hitachi, Ltd., Japan). The system operated at an acceleration voltage of 10 kV and a beam current of 100 pA. The exposed resist was developed in a 1:3 solution of methyl isobutyl ketone and isopropyl alcohol for 1 minute at room temperature. An 80 nm-thick Pr-Ge layer was subsequently deposited via electron-beam evaporation (KVE-E2000, Korea Vacuum Tech Co., Korea), followed by a lift-off process in acetone. Finally, a 100 nm-thick Au layer was deposited using a magnetron radio-frequency sputtering system (DDHT-LSH2, Daedong High Tech Co., Korea).

## Supplementary information

Supplementary Information for Sub-1-volt, reconfigurable Gires-Tournois resonators for full-coloured monopixel array

Supplementary Video 1

## Data Availability

The colour pixel images and dataset are available upon request.
