# Supplementary material for: Sub-1-volt, reconfigurable Gires-Tournois resonators for full-coloured monopixel array
Source: Light Sci Appl. 2026 Feb 28;15:134. doi: 10.1038/s41377-026-02228-2 (PMC12949994; doi:10.1038/s41377-026-02228-2)
Supplement: Supplementary file 1 — Supplementary Information for Sub-1-volt, reconfigurable Gires-Tournois resonators for full-coloured monopixel array [file 41377_2026_2228_MOESM1_ESM.docx]

**Supplementary Information for**

**Sub-1-volt, reconfigurable Gires-Tournois resonators for full-coloured monopixel array**

Joo Hwan Ko^1,2^†, Hyo Eun Jeong^1,6^†, Serim Kim^1^, Doeun Kim^1,3^, Se Yeon Kim^1^, Young Jin Yoo^2^, Hyeon-Ho Jeong^1,3,4^*****, Young Min Song^1,4,5,6^*****

^1^*School of Electrical Engineering and Computer Science, Gwangju Institute of Science and Technology, Gwangju 61005, Republic of Korea.*

^2^*Department of Mechanical Engineering, Massachusetts Institute of Technology, Cambridge, Massachusetts 02139, USA.*

*^3^GIST InnoCORE AI-Nano Convergence Initiative for Early Detection of Neurodegenerative Diseases, Gwangju Institute of Science and Technology, Gwangju 61005, Republic of Korea.*

^4^*Department of Semiconductor Engineering, Gwangju Institute of Science and Technology, Gwangju 61005, Republic of Korea.*

^5^*AI Graduate School,* *Gwangju Institute of Science and Technology,* *Gwangju 61005, Republic of Korea.*

^6^*School of Electrical Engineering, Korea Advanced Institute of Science and Technology, Daejeon 34141, Republic of Korea.*

†*J. H. Ko and H. E. Jeong contributed equally to this work.*

*****Corresponding author

E-mail: jeong323@gist.ac.kr; ymsong@kaist.ac.kr

**This PDF file includes:**

Supplementary Note 1 to 2

Figs. S1 to S44

Tables S1 to S6

Movie S1

References (1 to 61)

**Other Supplementary Materials for this manuscript include the following:**

Movie S1

Supplementary information accompanies the manuscript on *the Light: Science & Applications* website (http://www.nature.com/lsa)

Supplementary Note 1 Strategy of designing Gires-Tournois resonator with tri-layer structure to enhance sensitivity in optical modulation.

To enhance chromaticity, resonance based optical modulators for full colour generation should be configured to be sensitive to modulation in low refractive index medium. As a simple method for the dynamic display involves using a Gires-Tournois resonator structure in a low refractive index medium, designed using transmission line theory, described by the following equation:

$$\begin{aligned} Z_{in}=Z_{a}\frac{Z_{m}+iZ_{a}\tan\beta_{a}t_{a}}{Z_{a}+iZ_{m}\tan\beta_{a}t_{a}}\#\left( 1 \right) \end{aligned}$$

$Z_{in}$ is the input impedance of the system, where $\beta_{a}$ represents the wavenumber in the low refractive index medium, specifically active layer, given by ${2\pi n}_{a}/\lambda$. The active layer and metal reflector are associated with impedances $Z_{a}=Z_{0}/n_{a}$ and $Z_{m}=Z_{0}/N_{m}$, where $N_{m}=n_{m}+ik_{m}$, respectively. Here, $n_{m}$ and $k_{m}$ are refractive index and extinction coefficient of the metal, and $Z_{0}$ is the impedance of free space. Therefore, the reflection coefficient $r$ of incident light from air is given by

$$\begin{aligned} r=\frac{Z_{in}-Z_{0}}{Z_{in}+Z_{0}}\#\left( 2 \right) \end{aligned}$$

and when $R=1$, unity absorption is achieved, resulting in impedance matching, i.e., $Z=Z_{0}$, and the absorption $A=1-R=0$. To determine the values, Au layer was chosen as the metal reflector, and the low refractive index medium was set to approximately 1.5, similar to the refractive index of polyaniline (PANI). The impedance matching condition is then represented by a simple equation as a function of the dielectric layer thickness ($t_{a}$).

$$\begin{aligned} \tan\left( \frac{2\pi n_{a}t_{a}}{\lambda} \right)=i\frac{n_{a}\left( N_{m}+1 \right)}{N_{m}-n_{a}^{2}}\#\left( 3 \right) \end{aligned}$$

$$\begin{aligned} f\left( t_{a} \right)=tan \left( \frac{2\pi n_{a}t_{a}}{\lambda} \right)-i\frac{n_{a}\left( N_{m}+1 \right)}{N_{m}-n_{a}^{2}}\#\left( 4 \right) \end{aligned}$$

As shown by the simplified equation, the low refractive index medium contains only real values. Therefore, unity absorption cannot be achieved by varying thickness ($t_{a}$) if the imaginary part is not eliminated.

Lossy dielectrics with complex refractive indices produce significant phase shifts at the interface, making it challenging to predict the resonance condition. To address this, a low refractive index medium is required that can cancel the imaginary part through optical modulation. Consequently, the tri-layer structure is proposed as an improved design approach as shown in **Figs. S16a and S16b**.

$$\begin{aligned} Z_{in,l}=Z_{l}\frac{Z_{m}+iZ_{l}\tan\beta_{l}t_{l}}{Z_{l}+iZ_{m}\tan\beta_{l}t_{l}}\#\left( 5 \right) \end{aligned}$$

$$\begin{aligned} Z_{in,a}=Z_{a}\frac{Z_{in,l}+iZ_{a}\tan\beta_{a}t_{a}}{Z_{a}+iZ_{in,l}\tan\beta_{a}t_{a}}\#\left( 6 \right) \end{aligned}$$

$$\begin{aligned} r=\frac{Z_{in,a}-Z_{0}}{Z_{in,a}+Z_{0}}\#\left( 7 \right) \end{aligned}$$

In low refractive index materials, a reduction in complex refractive index due to thickness changes is not typically applicable to most lossy materials. To achieve unity absorption, the complex refractive index must be modified through effective material engineering, such as controlling porosity (**Fig. S17**). Therefore, we selected Pr-Ge to achieve the impedance matching condition, resulting in single absorption (**Fig. S16c**). As a result, the designed resonator is highly sensitive to optical modulation in the active layer.

The effective index of the material is calculated based on the volume averaging theory (VAT). The effective refractive index ($n_{eff}$) and effective extinction coefficient ($k_{eff}$) of two-phase nanocomposite material can be determined as follows:

$$\begin{aligned} n_{eff}^{2}=\frac{1}{2}\left[ A+\sqrt{A^{2}+B^{2}} \right]\#\left( 8 \right) \end{aligned}$$

$$\begin{aligned} k_{eff}^{2}=\frac{1}{2}\left[ -A+\sqrt{A^{2}+B^{2}} \right]\#\left( 9 \right) \end{aligned}$$

where

$$\begin{aligned} A=\emptyset\left( n_{d}^{2}-k_{d}^{2} \right)+\left( 1-\emptyset\right)\left( n_{c}^{2}-k_{c}^{2} \right)\#\left( 10 \right) \end{aligned}$$

$\begin{aligned} B=2n_{d}k_{d}\emptyset+2n_{c}k_{c}\left( 1-\emptyset\right)\#\left( 11 \right) \end{aligned}$The $n_{d}$ and $n_{c}$ represent the refractive indices of the continuous and dispersed phases, respectively, while $k_{d}$ and $k_{c}$ signify the extinction coefficients. The porosity is denoted by $\emptyset$. In this case, the dispersed phase is considered as vacuum, resulting in $n_{d}=1$ and $k_{d}=0$.

# Supplementary Note 2. Wavelength tuning under negative group delay conditions with various redox states of PANI.

By utilizing different redox states of PANI, the wavelength can be adjusted under negative group delay conditions in the *r*-GT structure, leading to significant shifts in the reflective wavelength. The *r*-GT configuration induces significant changes in the group velocity of light, enabling the observation of slow, fast, and negative group velocity phenomena. Negative group delay, where a light pulse appears to exit the medium before entering, is achieved through the resonant trapping of certain wavelengths within the spacer layer between the upper reflective surface and the underlying high reflector as shown in **Fig S18**.

The refractive index and extinction coefficient of PANI vary with its redox state, enabling it to act as a tunable upper-surface reflector and leading to changes in resonance conditions (**Fig. S3**). In **Fig. 1f**, the scattered dots indicate the changes in the complex refractive index of PANI under different redox states. Compared to other configurations, the simulated reflectivity of the *r*-GT resonator reveals that the Pr-Ge structure effectively optimises resonance conditions by fine-tuning its refractive index. **Fig. 1g** shows reflectivity changes for different configurations: ⅰ) asymmetric Fabry–Pérot (F.-P.) resonator without Ge, where PANI is deposited directly onto the Au substrate; ⅱ) Trilayer Gires-Tournois (GT) resonator with a-Ge incorporating a dense amorphous Ge layer inserted between Au and PANI; and ⅲ) Tailored trilayer GT with Pr-Ge, consisting of a porous Ge layer between Au and PANI. Among these, the Pr-Ge structure exhibits the most pronounced resonance shift during the redox transition of PANI, indicating enhanced optical modulation performance.


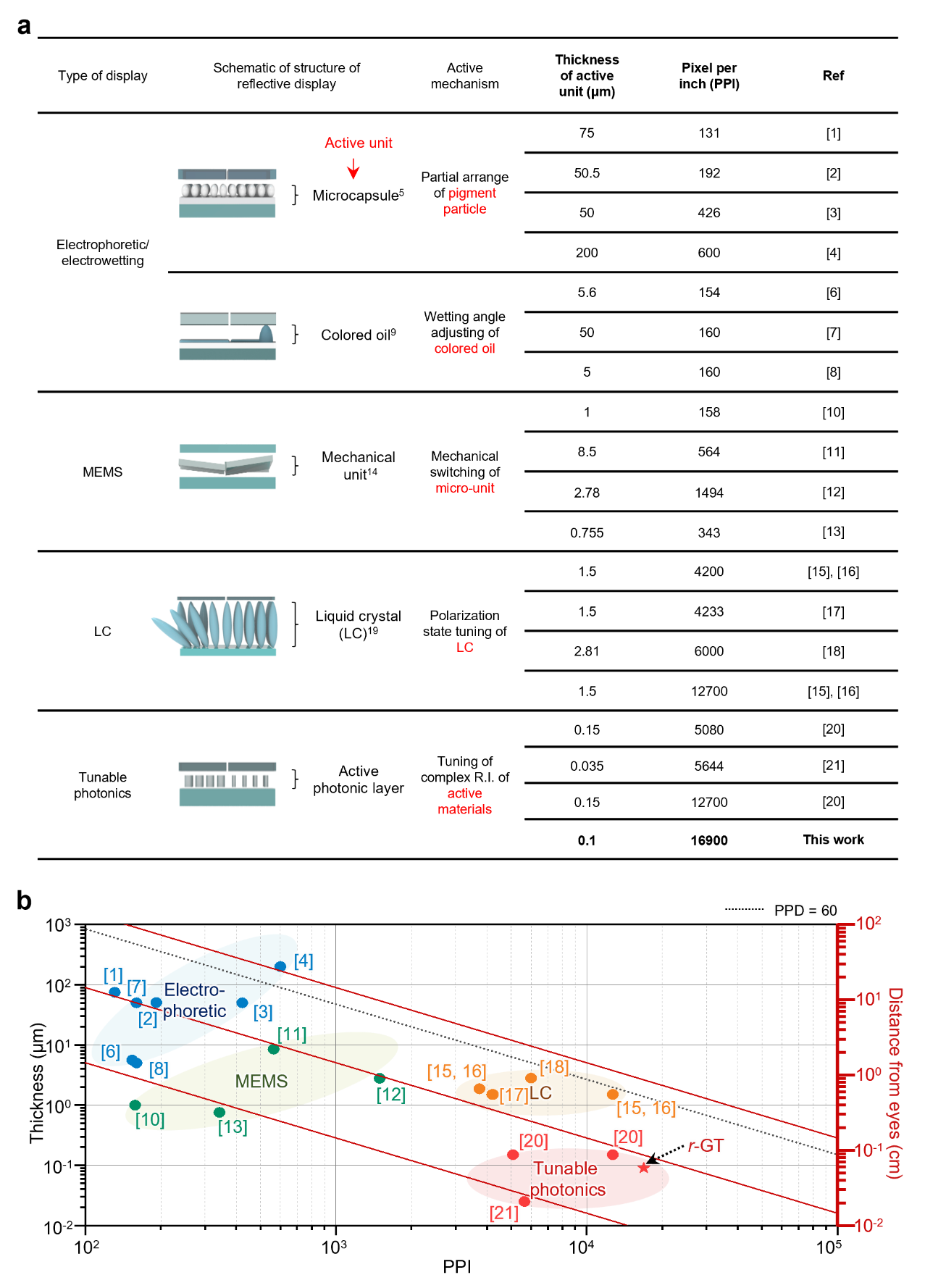
Fig. S1. System construction, the thickness of active layer, dimension, and volume of the conventional reflective display. (a-b) Comparison of the structures of various commercial reflective displays with their corresponding active layer and pixel density. Electrophoretic displays*^1-5^* and electrowetting displays*^6-9^* adjust reflectivity using charged pigment particles encapsulated in microcapsules and hydrophobic-coated polar solvents, respectively. MEMS-based displays*^10-14^* rely on the mechanical switching of micro-units to manipulate external light reflection LC displays*^15-19^* adjust the polarization state of liquid crystals under an electric field to modulate ambient light passing through colour filters. These commercial reflective display structures are limited in thickness and pixels per inch (PPI) due to their specific mechanisms. In contrast, tunable photonics*^20, 21^* allows for high pixel density with a thin structure, with the Gires-Tournois (*r*-GT) resonator achieving 16,900 PPI.


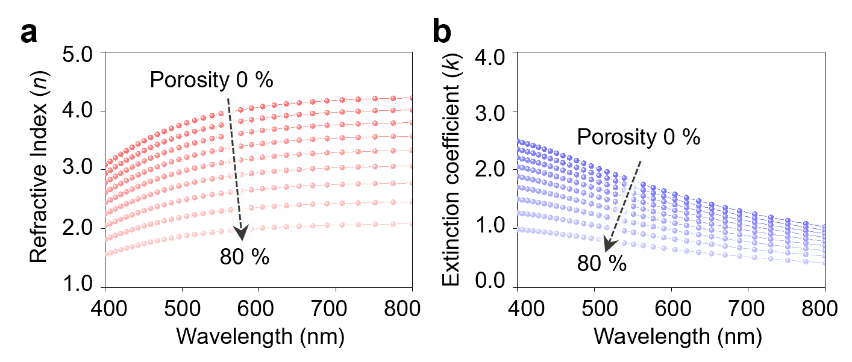
Fig. S2. Wavelength-dependent refractive index (*n*, a) and extinction coefficient (*k*, b) of Pr-Ge as a function of porosity (Pr).


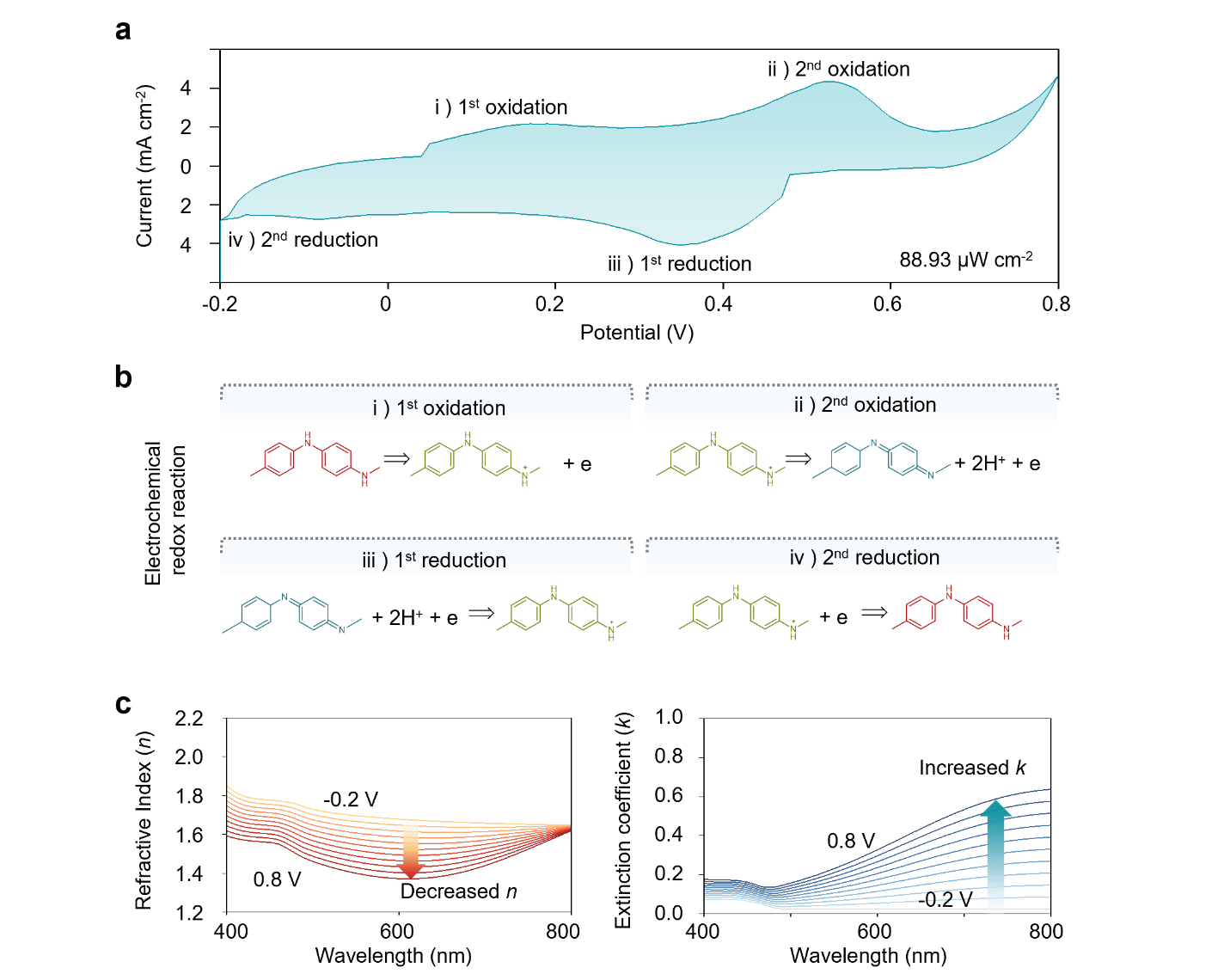
Fig. S3. The cyclic voltammetric (CV) curve of polyaniline (PANI). (a) The CV curve of PANI which shows two pairs of redox reactions, with the corresponding average power density. Each cycle sweeps the voltage from -0.2 V to 0.8 V (scan rate: 0.05 V s^-1^), consuming 88.93 µW cm^-^² of power. The redox process includes two oxidation peaks (i, ii) and two reduction peaks (iii, iv). (b) The electrochemical activation by a pair of electrons and protons induces modulation of its oxidation state, resulting in structural changes in the PANI molecule. (c) Modulation of the refractive index (*n*) and extinction coefficient (*k*) according to the applied potential, decreasing and increasing respectively with changes in the optical properties. The insulating state (-0.2 V) and semiconducting state (0.8 V) were directly measured using an ellipsometer (Elli-SEU, Ellipsotech, South Korea) from the electrodeposited PANI thin film on an Au substrate, and intermediate values were calculated through continuous interpolation.


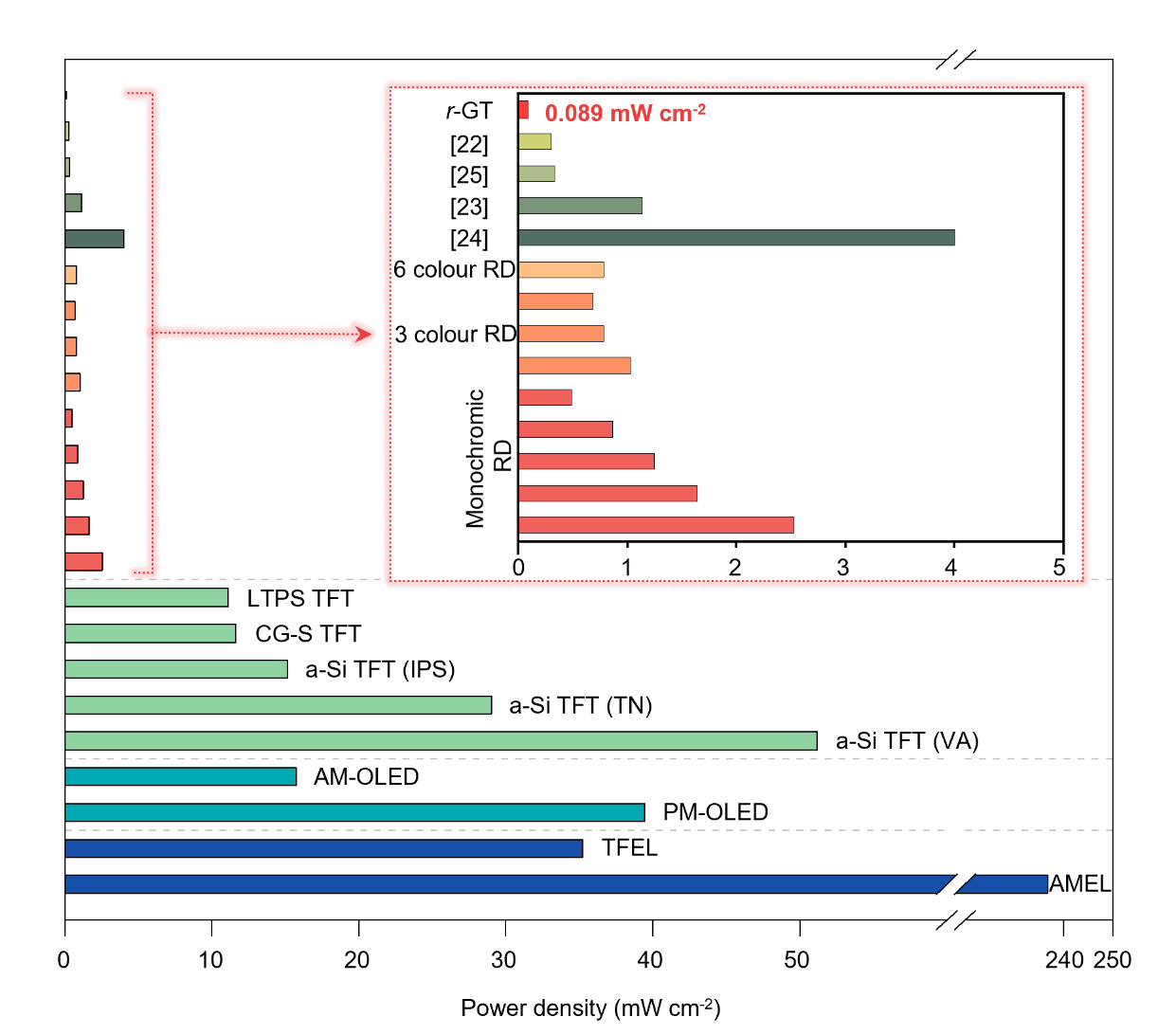
Fig. S4. Power density of various displays*^22-26^*. Power density of displays. Reflective display (RD), liquid crystal (LC): low-temperature polycrystalline silicon (LTPS), thin film transistor (TFT), continuous grain silicon (CGS), amorphous silicon (a-Si), in-plane switching (IPS), super-twisted Nematic types (STN), vertical alignment(VA), organic light-emitting display (OLED): passive matrix (PM), active matrix (AM), electroluminescent display (ELD): thin-film electroluminescent (TFEL), active-matrix electroluminescence (AMEL). The inlet graph compares the power density of the reflective display with *r*-GT resonator, which has a power density of 0.089 mW cm^-2^. The monochromic RD displays black and white, while the 3-colour RD additionally provides red, and the 6-colour RD includes multiple colours (red, green, blue, and yellow). Compared to recently reported electrochromic devices relying on ion doping and de-doping, *r*-GT operates with low power consumption.

#
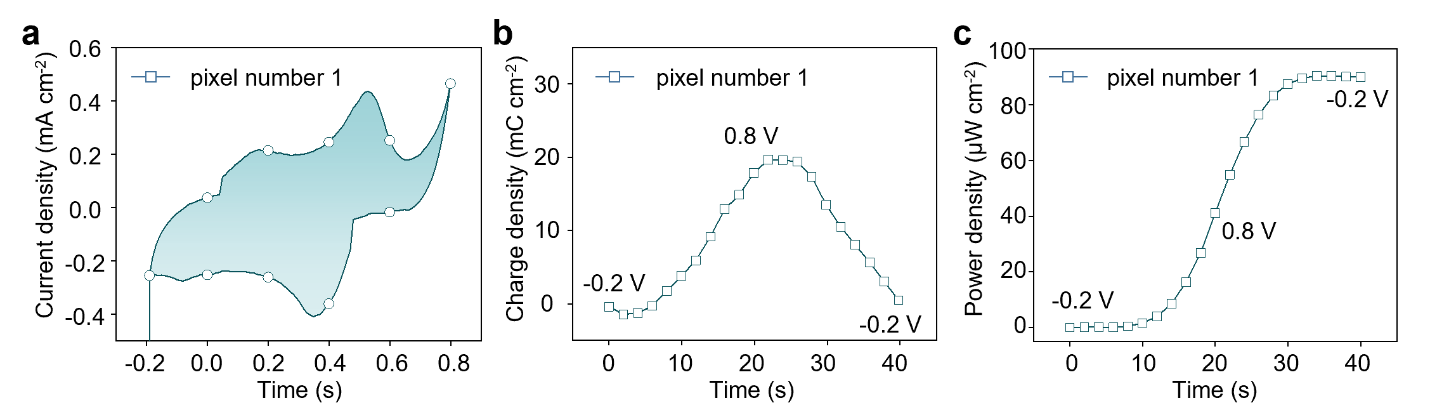
Fig. S5. Electrochemical performance of the *r*-GT monopixel during a single cycle. (a) CV curve of *r*-GT monopixel in the addressable monopixel array. (b) Reversible modulation of charge density during voltage sweep from -0.2 V to 0.8 V. (c) Calculated power density consumed by *r*-GT resonator per cycle.

#
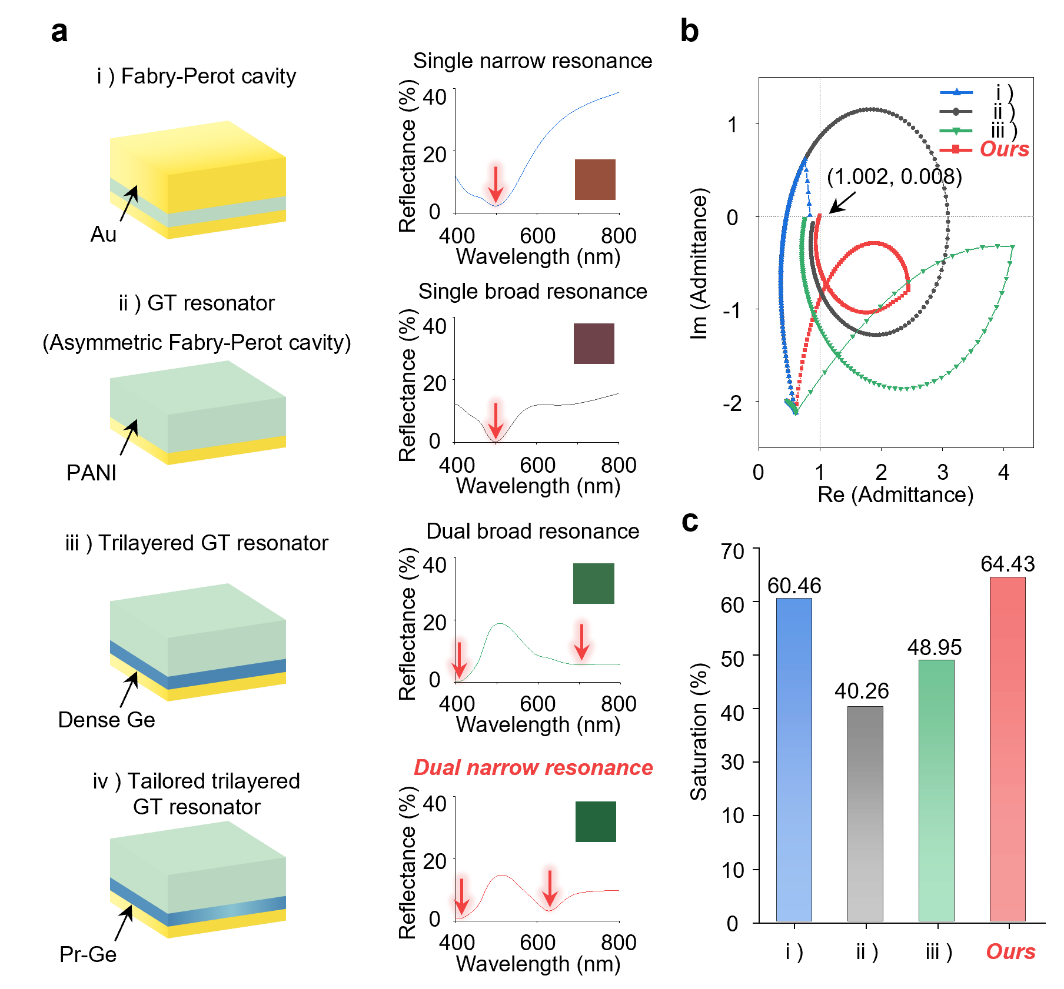
Fig. S6. Optical design strategies for enhanced colour quality. (a) Schematics of photonic structures and their reflection spectra: i) Fabry-Parot cavity, ii) GT resonator, iii) trilayered GT resonator, and iv) Tailored trilayered GT resonator (ours). (b) Calculated admittance diagram for i) to iv) at each resonance wavelengths. (c) Calculated saturation corresponding to (a). Both trilayered GT resonators enable additive colour generation in the visible range via dual resonance. However, design iv) with optimised porosity produces a deeper resonance dip, enhancing colour saturation.

#
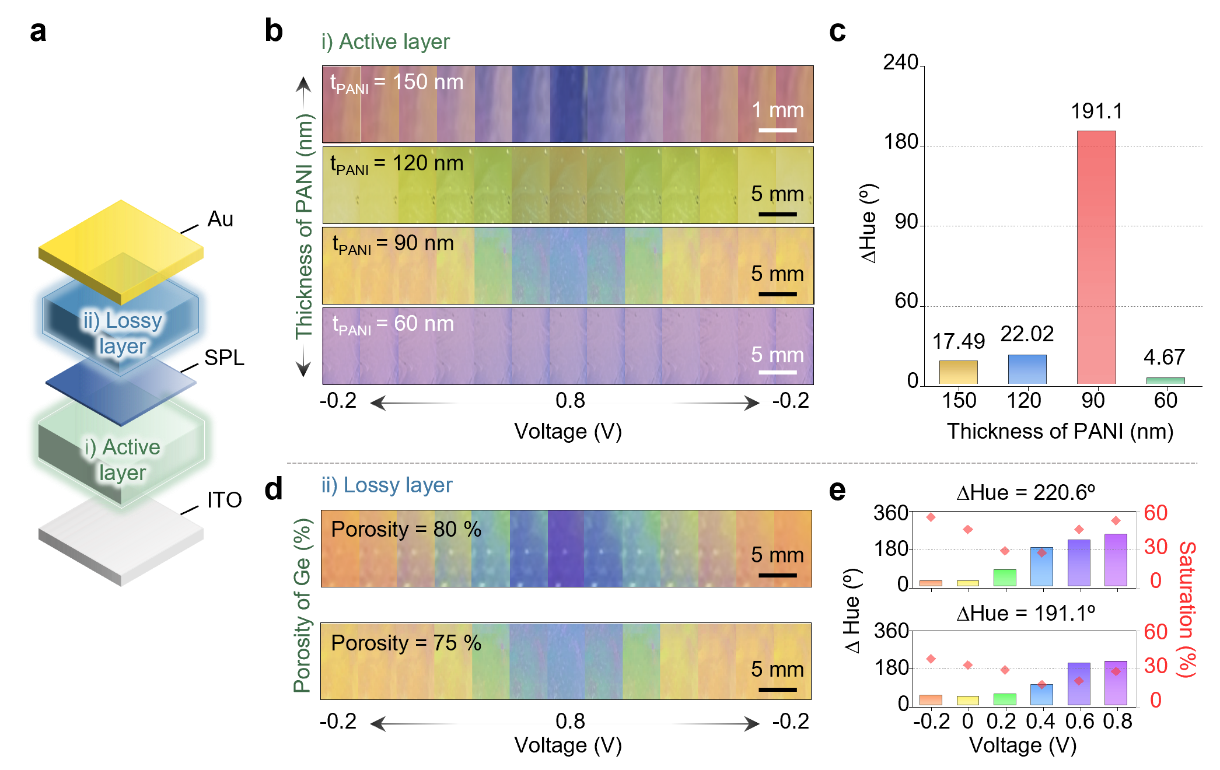
Fig. S7. Optical design optimization for enhanced hue range. (a) Schematic illustration of each layer comprising the *r*-GT resonator. (b) Photograph of reversible colour modulation corresponding to different thickness of active layers. (c) Colour modulation range of the *r*-GT structures as a function of PANI thickness (150 nm, 120 nm, 90 nm and 60 nm). (d) Photographic images illustrating the colour changes of *r*-GT resonators with different porosities under varying applied voltages. (e) Comparison of the hue range and saturation values corresponding to the images in (d).

#
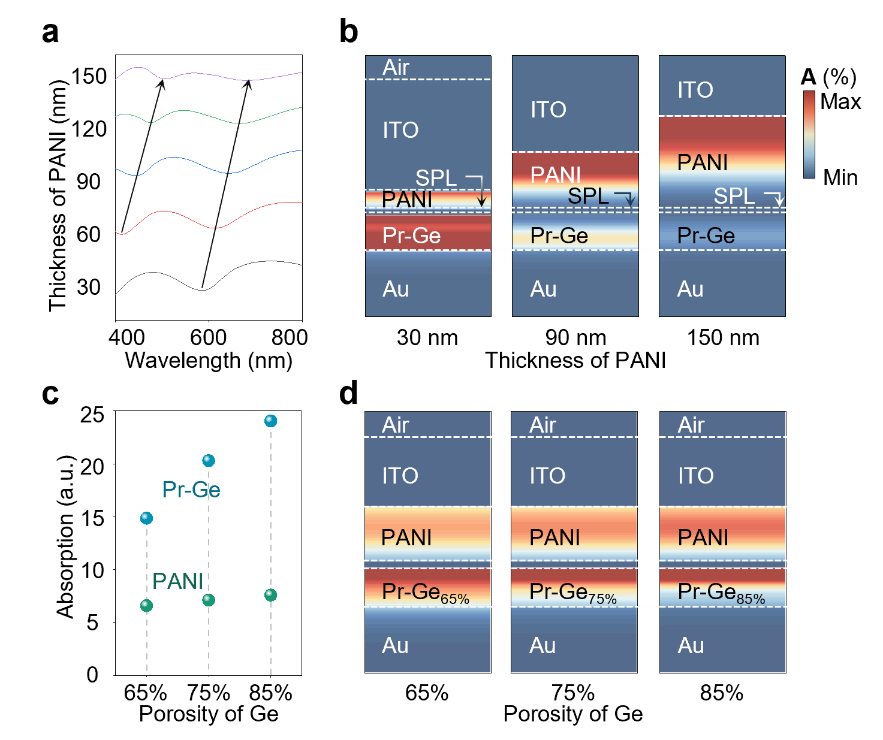
Fig. S8. Optical design optimization for enhanced hue range. Optimization strategy for dual-resonance spectral characteristics. (a) Calculated reflectance spectra with varying PANI thickness in the visible range. (b) Simulated absorption profiles at the resonant wavelengths for active layer thicknesses of 30 nm, 90 nm, and 150 nm. (c) Calculated absorption in each layer (Pr-Ge and PANI) as a function of the porosity of the lossy medium. (d) Simulated absorption profiles at the resonant wavelength for different porosities (Pr; 65%, 75% and 85 %).

#
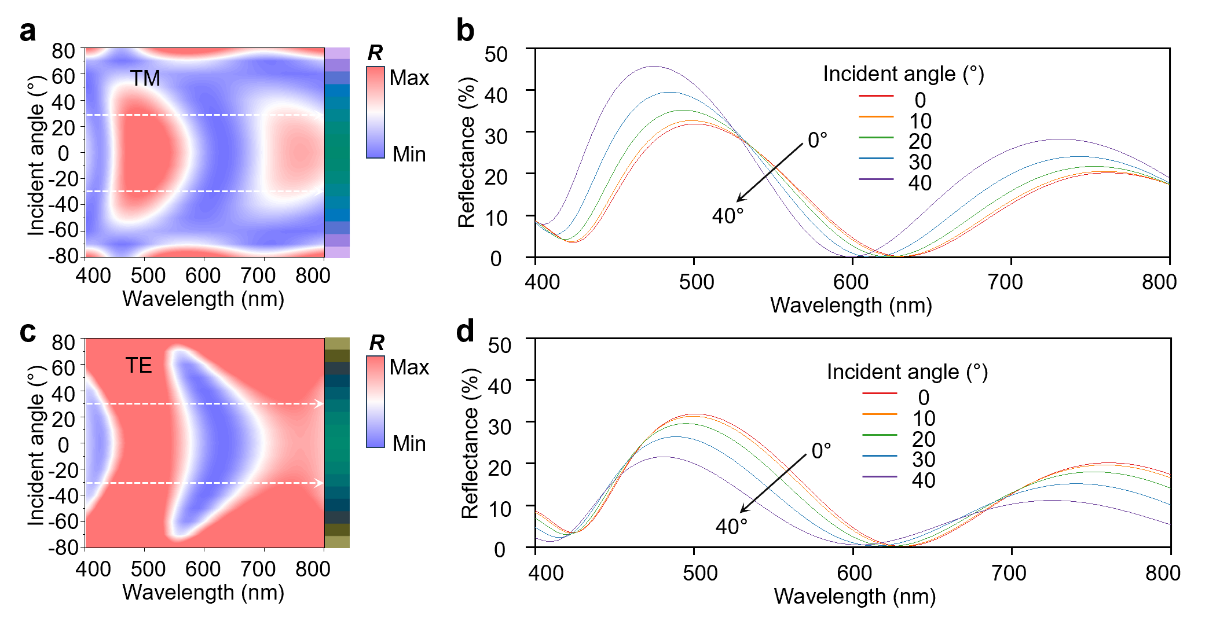
Fig. S9. Angular dependency of *r*-GT resonator. (a, c) Contour maps of reflectance under transverse magnetic (TM, a) and transverse electric (TE, c) polarizations, along with the corresponding reflected colours as a function of the incident angle ranging from -80° to 80°. The device exhibits stable colour performance within the angular range of -30° to 30°, demonstrating its high angular stability. (b, d) Reflectance spectra in the visible range measured at the incident angle ranging from 0° to 40°.

#
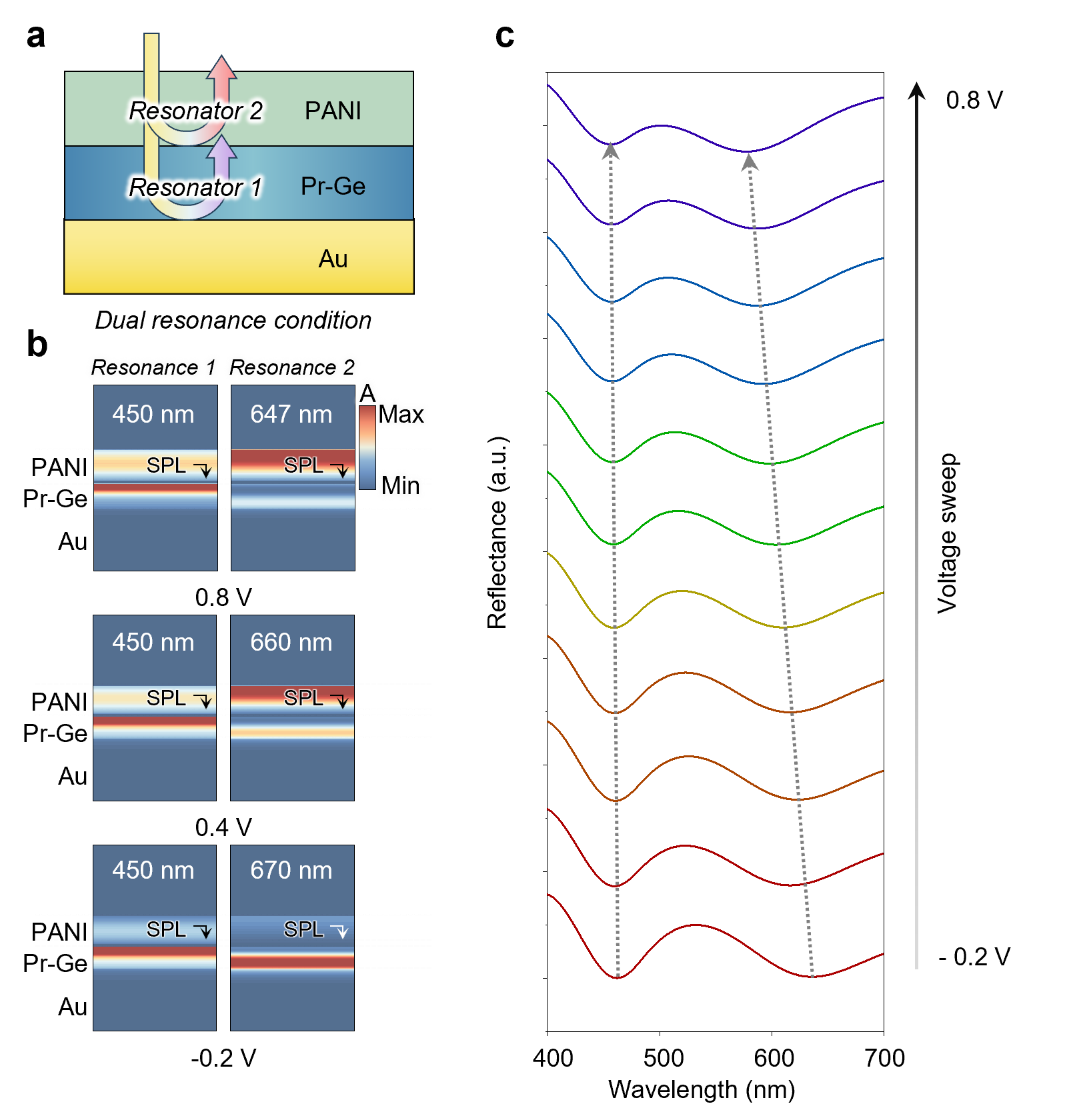
Fig. S10. Dual resonance of *r*-GT. (a) Illustrations of the *r*-GT resonator under dual resonance conditions, showing the position of each resonance. (b) Absorption profiles of *r*-GT monopixel at -0.2 V, 0.4 V, and 0.8 V. Under the reduced state (-0.2 V), the absorption in the PANI layer at the resonant wavelength decreases. (c) Measured reflection spectra of *r*-GT in metallic state (0.8 V) and semiconducting state (-0.2 V) of PANI with voltage sweep at the corresponding visible range.

#
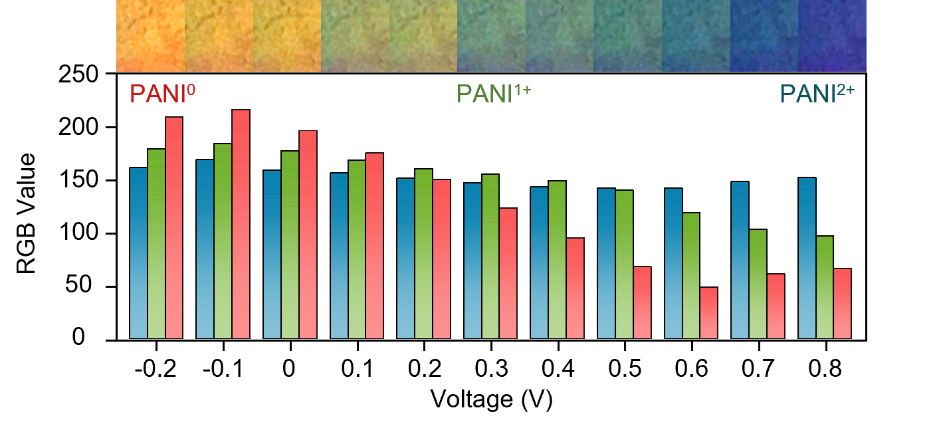
Fig. S11. RGB value variation of the *r*-GT resonator as a function of applied potential. Electrochemically induced RGB variation of the *r*-GT resonator under applied bias. The chemical state at -0.2 V corresponds to the fully reduced state (PANI^0^) of PANI, while that at 0.8 V represents the fully oxidized state (PANI^2+^).

#
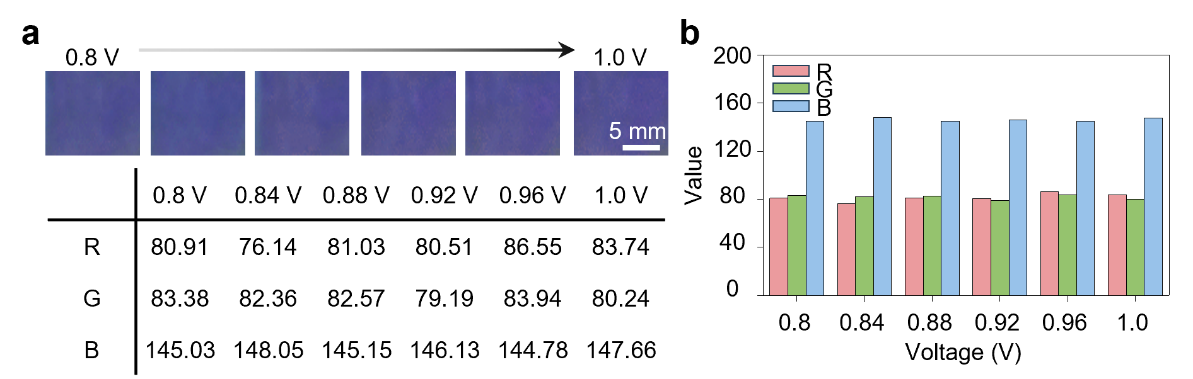
Fig. S12. Electrochromic colour shift and RGB analysis. (a) Colour changes under applied voltage range from 0.8 V to 1.0 V and (b) corresponding RGB values.

#
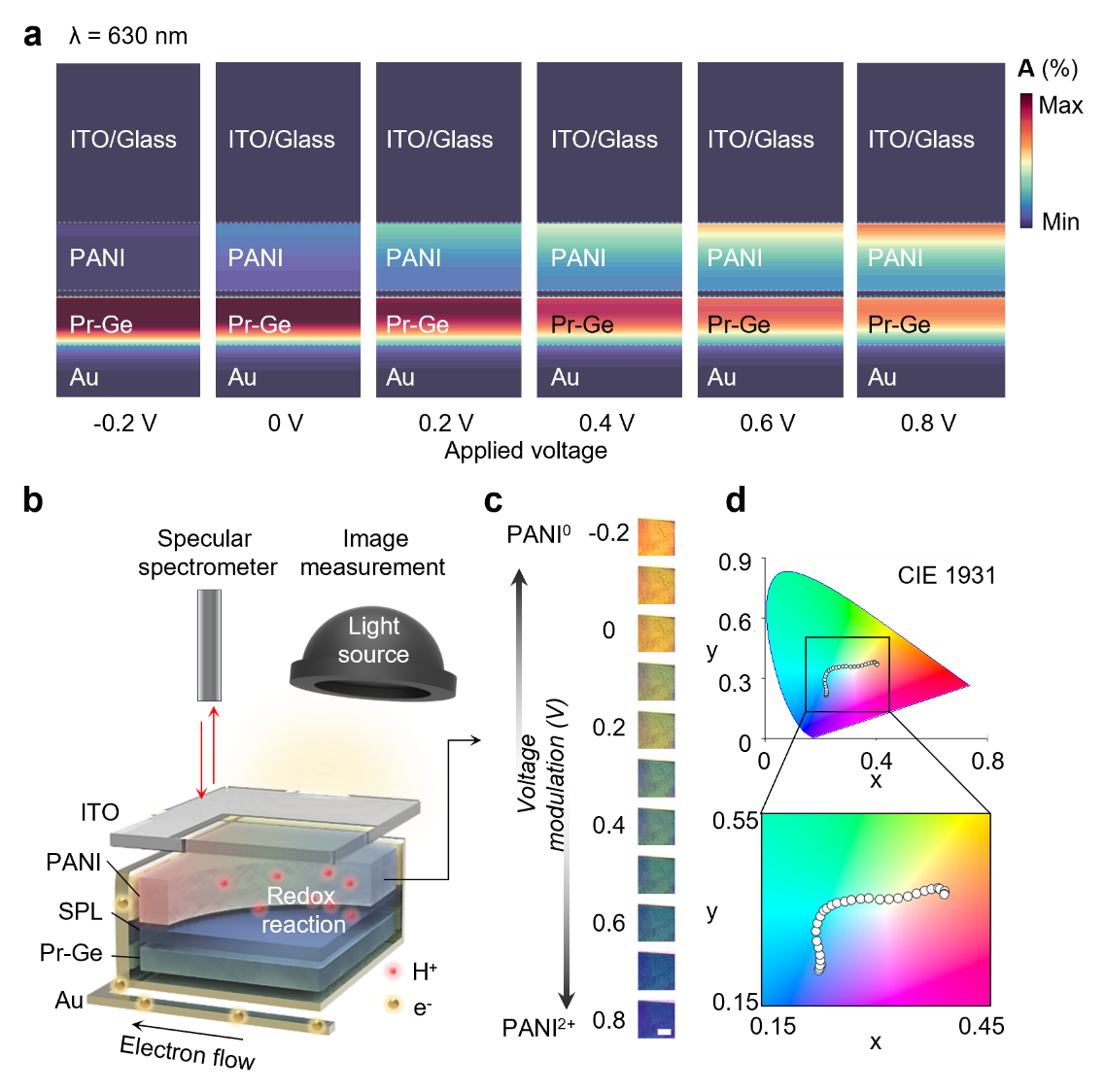
Fig. S13. Optical simulation and experimental validation. (a) Rigorous coupled wave analysis (RCWA) was employed to simulate the optical behaviour of *r*-GT under a plane wave of incident light. The simulation adjusted the complex refractive indices in accordance with the potential depicted in Fig. S2. The absorption profile across a cross-section of *r*-GT, highlighting variations of absorption tendency induced by different applied potentials on PANI. (b) Schematic of the active modulation of *r*-GT, accompanied by real-time measurements of the spectrum. (c) Photographs showing the colour modulation of the *r*-GT resonator, demonstrating a continuous change from red to purple in response to an applied potential ranging from -0.2 V to 0.8 V. (d) Chromaticity plots on the CIE coordinates extracted from the measured spectra, illustrating colour changes corresponding to the varying redox states of PANI.

#
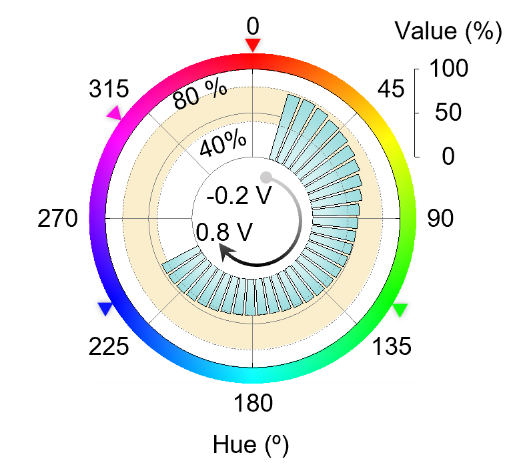
Fig. S14. Voltage-dependent colour modulation characteristics of the *r*-GT monopixel. Polar plot of the reflected hue and value extracted from the measured spectra as a function of applied voltage. The device maintains a value (brightness) above 40% across a wide hue modulation range (220.6°).

#
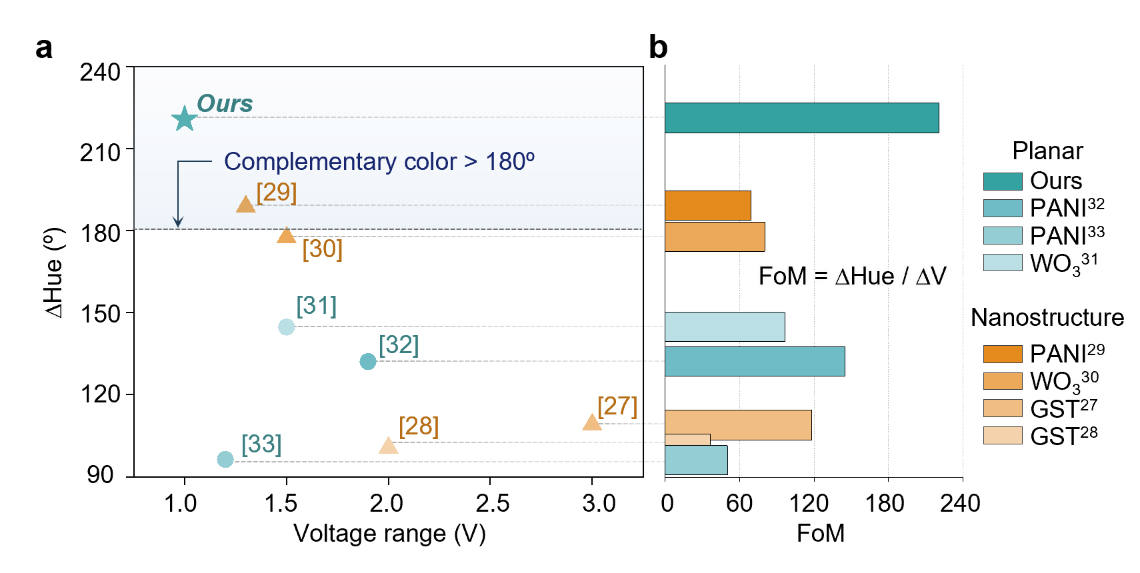
Fig. S15. Comparison of colour modulation range and voltage range in electrochromic resonators*^27-33^*. (a) Comparison of hue range and applied voltage range. The blue box means colour modulation range exceeding complementary colours (> 180º). (b) Comparison of the figure of merit (FoM = ΔHue / ΔV) between this device and reported electrochromic resonators.

#
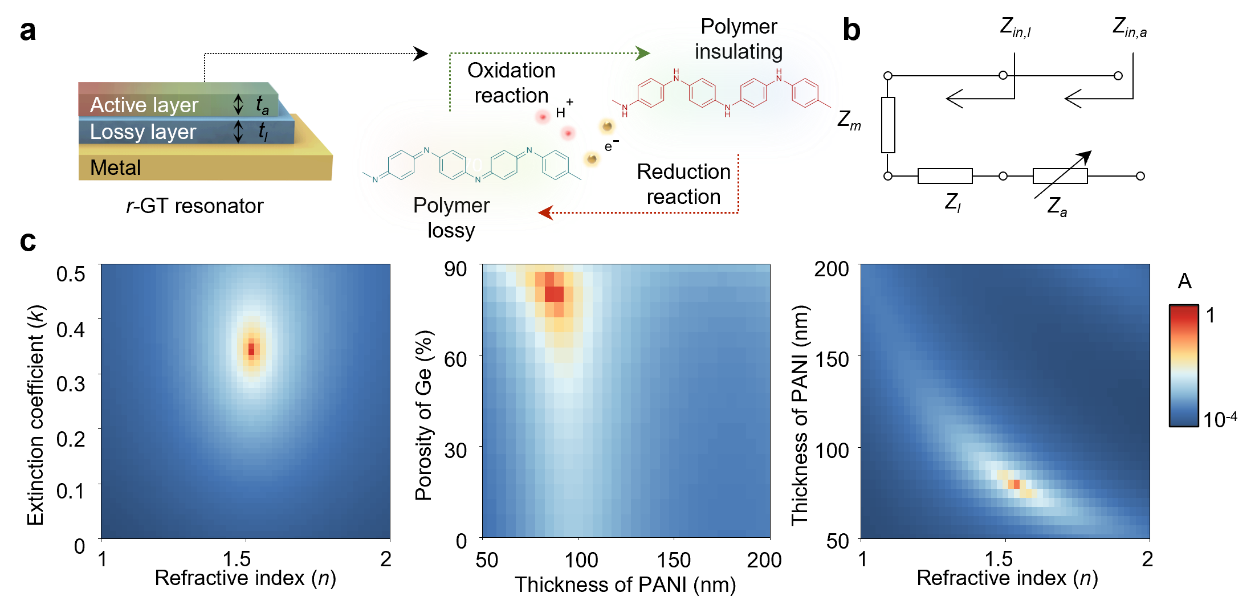
Fig. S16. Design of *r*-GT resonator for dynamic colour variation. (a) Schematic illustration of the tri-layered optical resonator, where the complex refractive index of PANI changes through a redox reaction. (b) Equivalent transmission line model of the tri-layered reconfigurable GT structure. The arrow indicates active modulation of the impedance in the active layer (PANI, *Z_a_*). *Z_l_* and *Z_m_* represent impedance of lossy layer and metal layer, respectively. (c) Absorption characteristics of the proposed design. Absorptance contour plots for varying thickness of PANI and porosity of Ge under resonant wavelength.

#
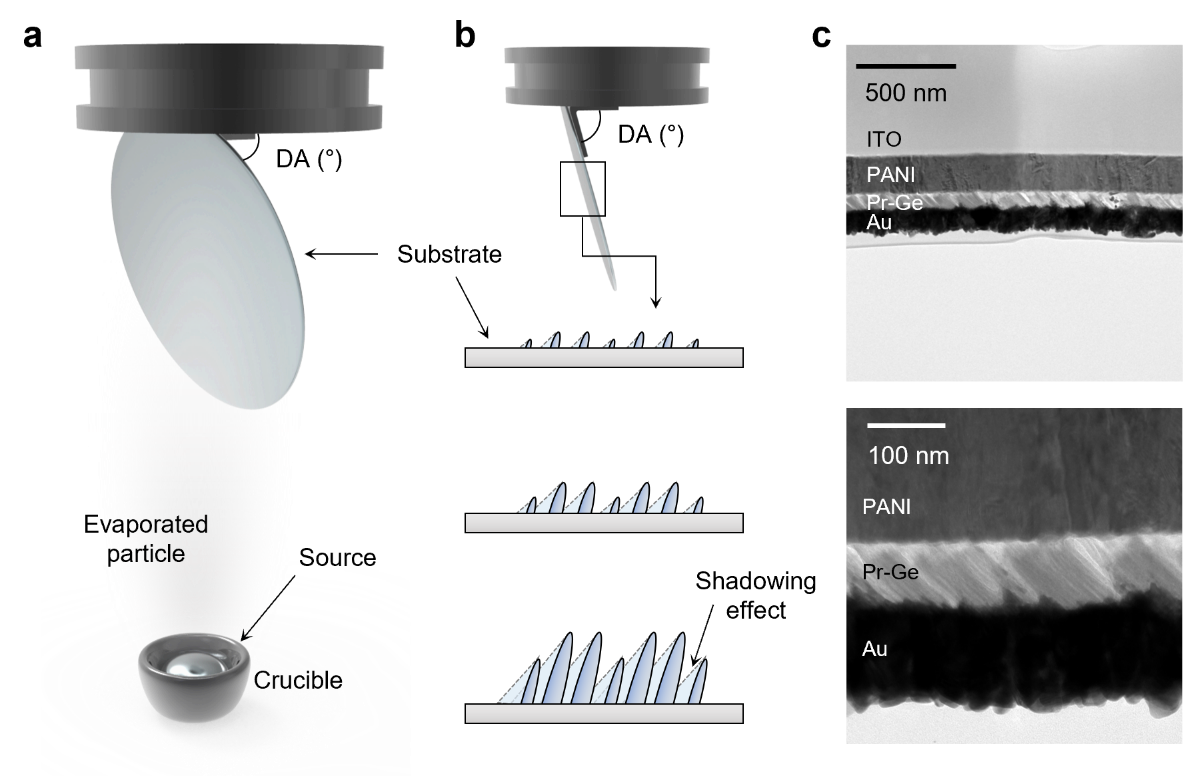
Fig. S17. Optical effect of porosity variation. (a) Schematic of experimental setup with deposition angle (DA). (b) Glancing angle deposition process of porous medium through the shadowing effect, resulting in modulation of the effective index as a porosity change. (c) Transmission electron microscopy (TEM) image of the deposited morphology of Ge layer to modulate the porosity via e-beam evaporation. Scale bars are 500 nm (upper) and 100 nm (bottom).

#
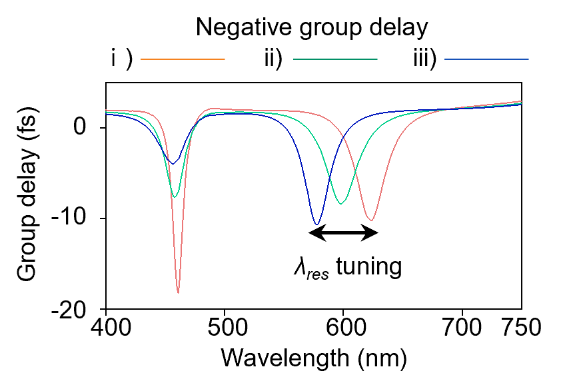
Fig. S18. Group delay response of *r*-GT across different voltage sweep ranges for various resonator configuration. i) asymmetric F.-P. cavity, ii) Trilayer GT resonator, and iii) Tailored trilayer GT resonator.

#
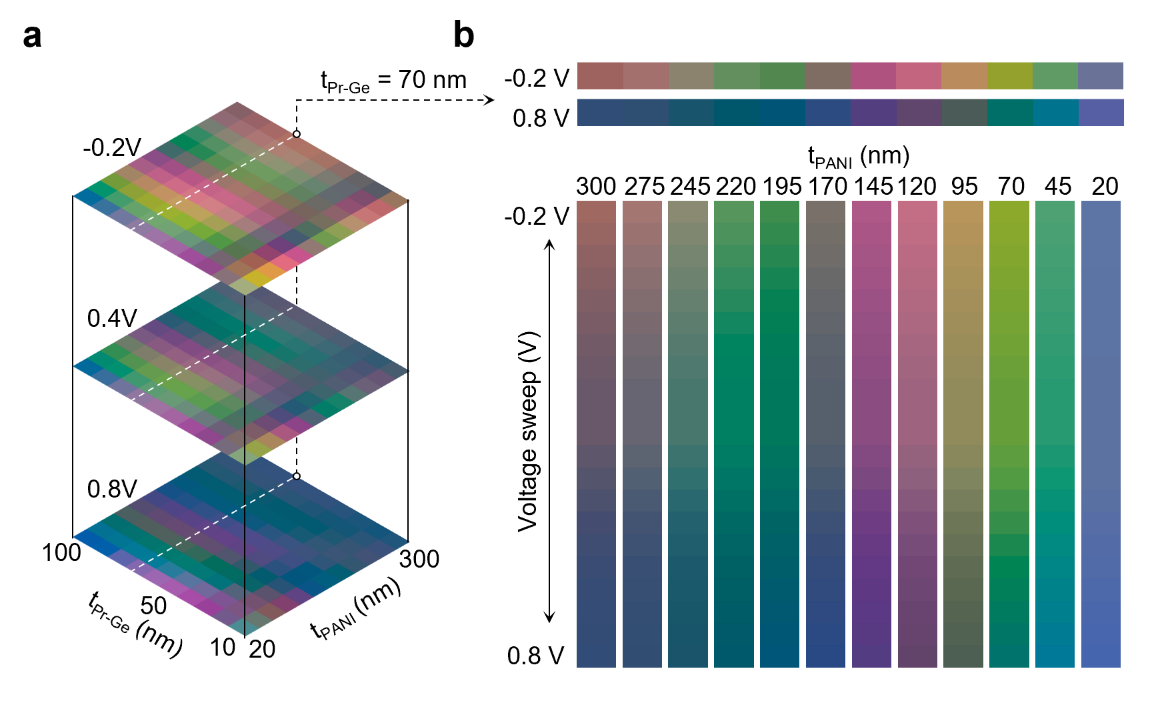
Fig. S19. Colour modulation in tailored trilayer GT resonators. (a) Voltage-dependent reflectance colour charts of tailored trilayer GT resonators (Au/Pr-Ge/PANI/ITO) as a function of Pr-Ge and PANI thicknesses. (b) Representative colour palette is shown for a fixed Pr-Ge thickness of 70 nm, with variation in PANI thickness and applied voltage.

#
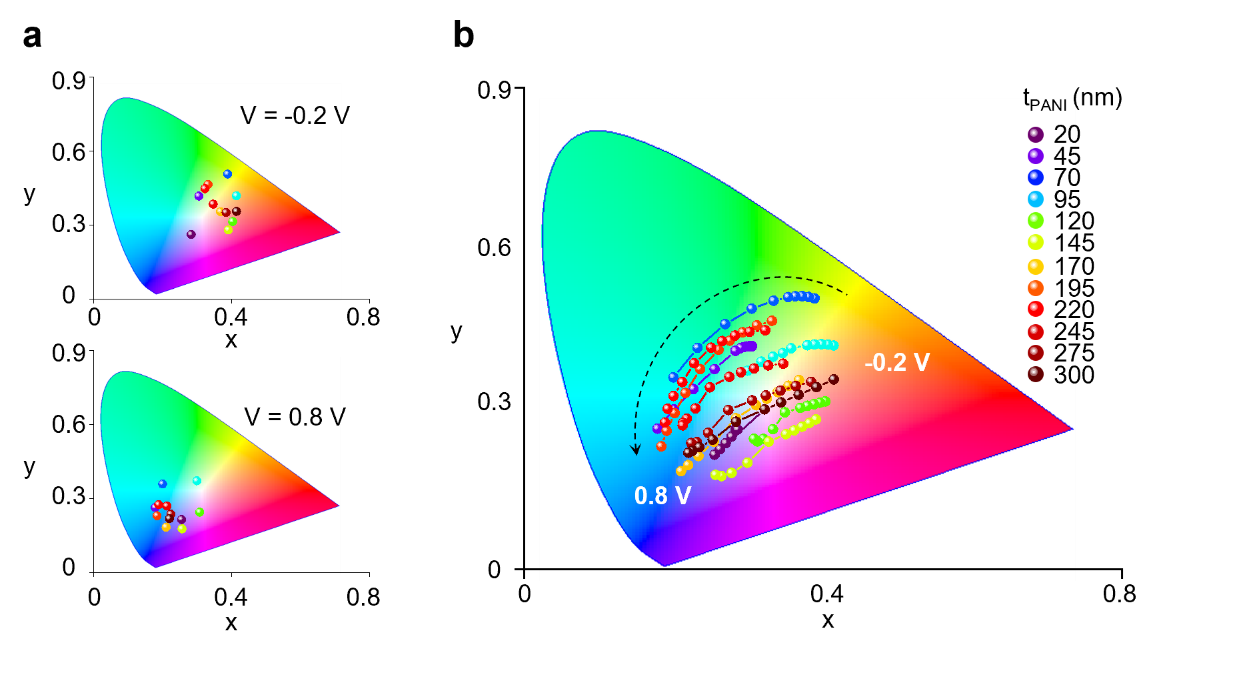
Fig. S20. CIE 1931 chromaticity coordinates of the *r*-GT resonator under various applied potentials. (a) Chromaticity coordinates and sRGB coverage at fully reduced (-0.2 V) and fully oxidized (0.8 V) states for different PANI thicknesses. (b) CIE colour coordinates for *r*-GT resonator with varying PANI thickness under applied voltage range -0.2 V to 0.8 V in steps of 0.1 V.

#
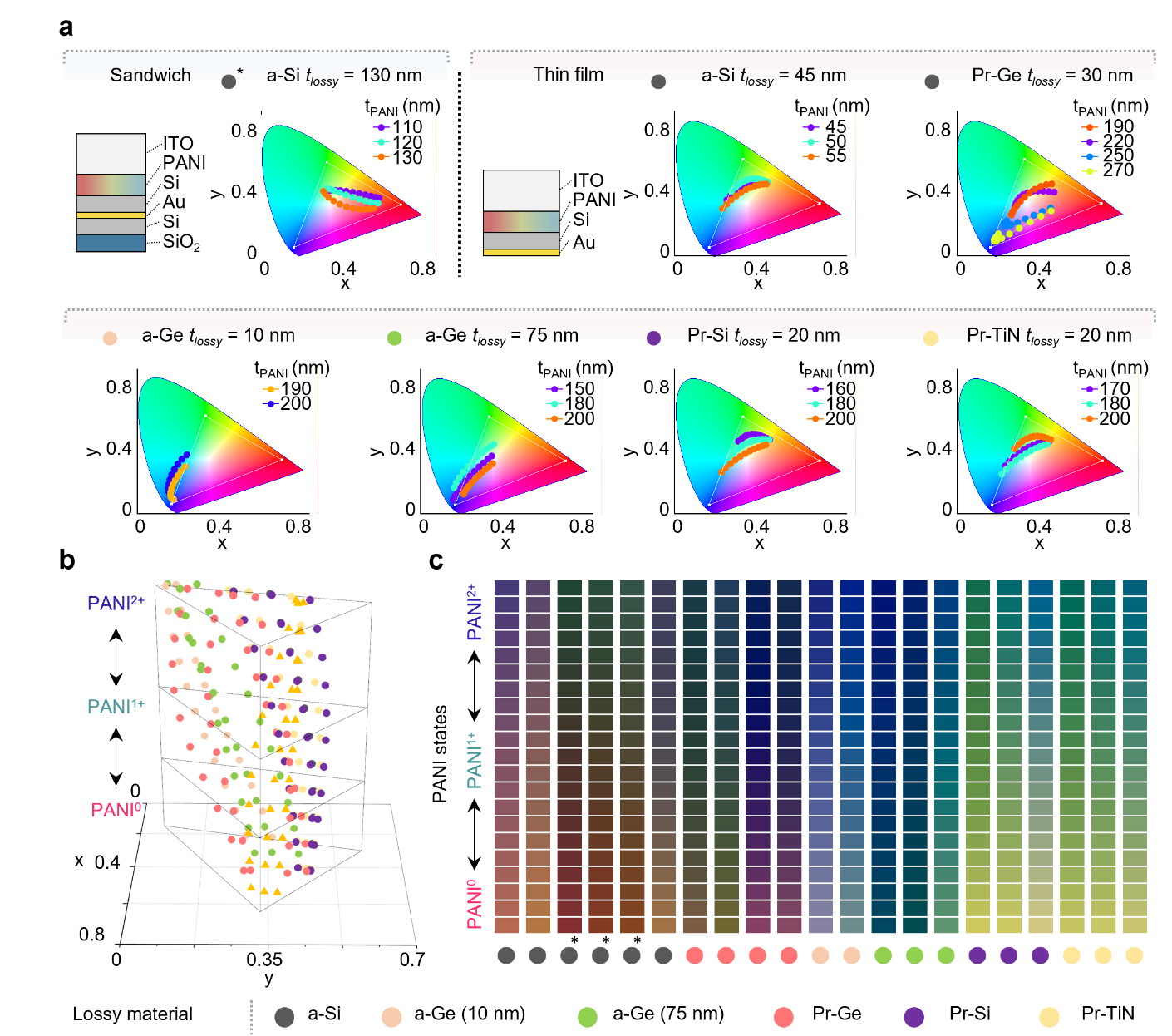
Fig. S21. *r*-GT design and optical simulation result for diverse colouration. (a) Two types of *r*-GT structures utilizing various lossy materials. In particular, a sandwich structure is shown to generate a distinct red colour by incorporating an additional lossy or dielectric layer. Furthermore, lossy materials with different complex refractive indices are introduced to achieve diverse colour variations. (b) Chromaticity plots on the CIE coordinates, illustrating colour changes corresponding to the varying redox states of PANI. (c) A colour palette depicting the material combinations, represented with coloured symbols.

#
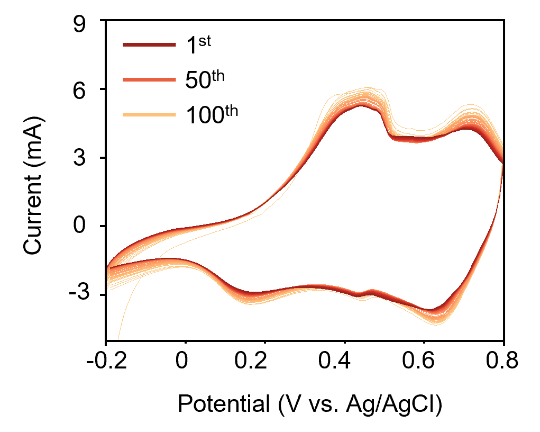
Fig. S22. Electrochemical stability of the *r*-GT resonator. The *r*-GT monopixel demonstrates stable redox reaction during 100 cycles.

#
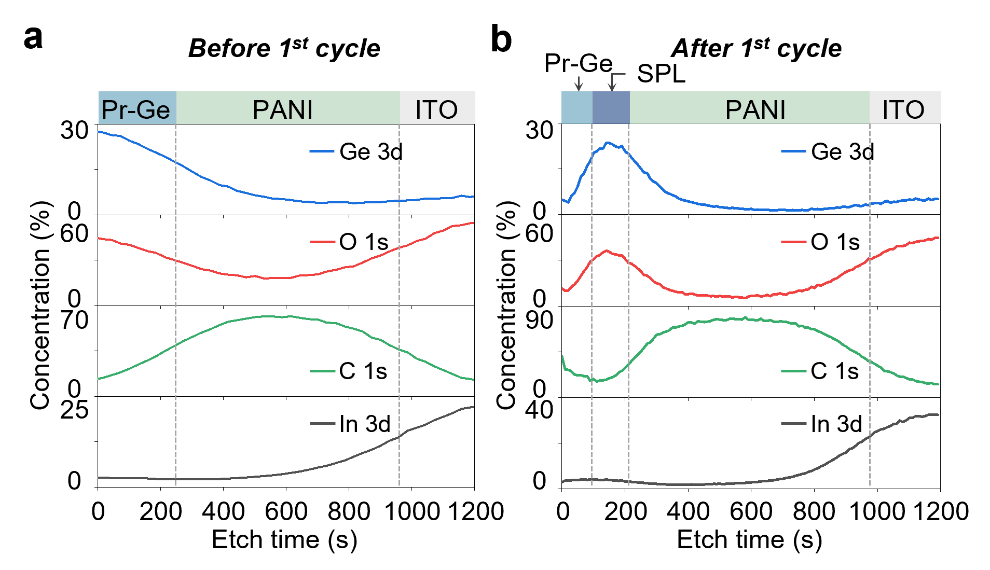
Fig. S23. The measured XPS depth profiles of before and after 1st cyclic voltammetry. (a) XPS depth profile of the Pr-Ge/PANI/ITO structure before the first redox reaction. (b) XPS depth profile of the *r*-GT resonator after the initial oxidation, showing SPL formation. For redox operation, an Au layer was deposited to protect the Pr-Ge layer and act as a reflective electrode; following the redox process, the Au was removed via etching prior to XPS depth profiling. An increased oxygen concentration is observed at the PANI/Pr-Ge interface following the initial oxidation, indicating the formation of GeO_2_ layer as SPL. The C 1s signal originates from the conducting polymer backbone structure, while the In 3d signal is attributed to the indium tin oxide (ITO) layer.

#
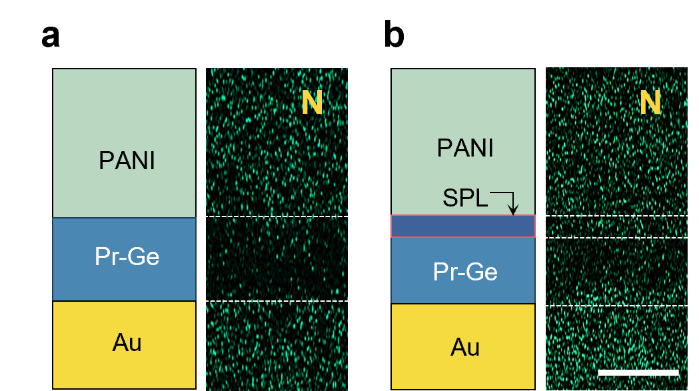
Fig. S24. EDS mapping of nitrogen before (a) and after (b) the first oxidation process. The elemental profile at the PANI/Pr-Ge interface shows an increased nitrogen concentration (green), attributed to doping and dedoping processes.

#
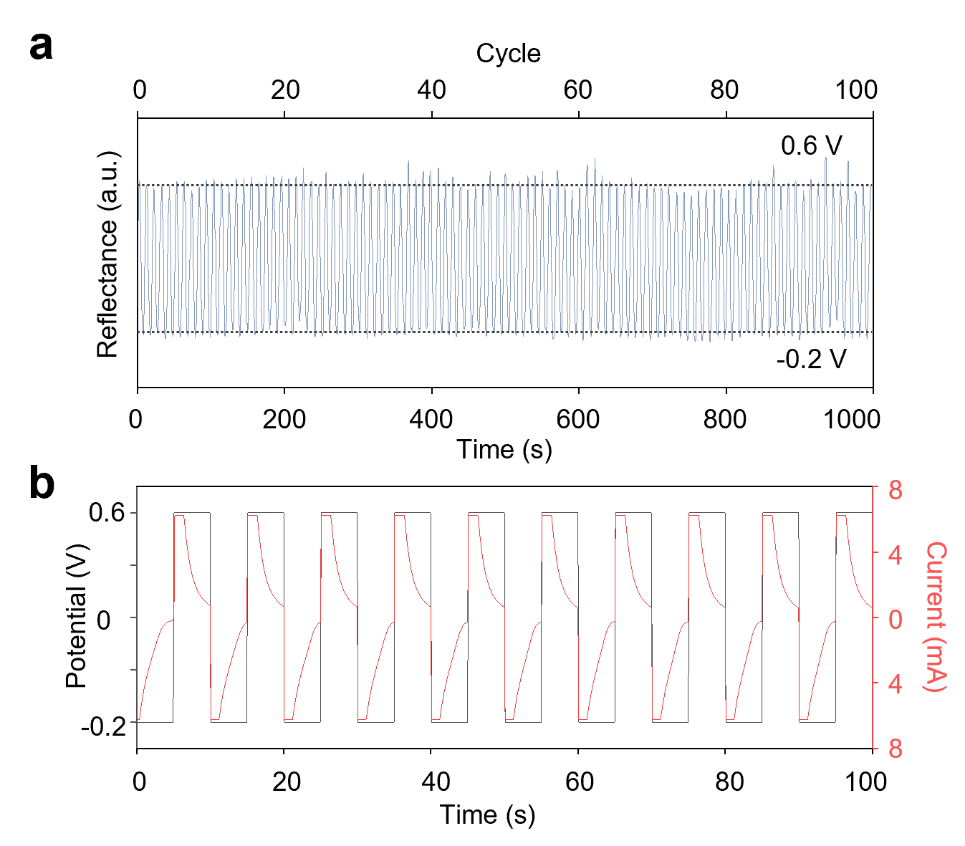
Fig. S25. Optical reversibility of *r*-GT resonator. (a) Reflectance reversibility with bias voltage cycled from -0.2 V to 0.6 V (interval: 10 s) for 100 cycles. (b) Measured current with applied voltage cycled from -0.2 V to 0.6 V for 10 cycles.

#
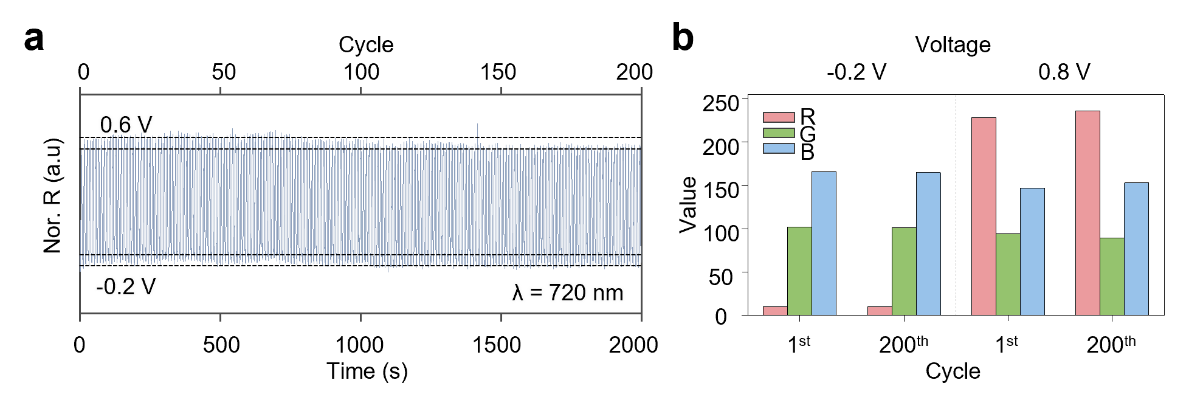
Fig. S26. Comparison of RGB values before and after 200 cycles. (a) Reflectance switching analysis over 200 cycles (interval: 10 s) performed on the same sample as in Fig. S21. (b) Calculated colour information based on the reflectance spectra measured at the first and 200^th^ cycles.

#
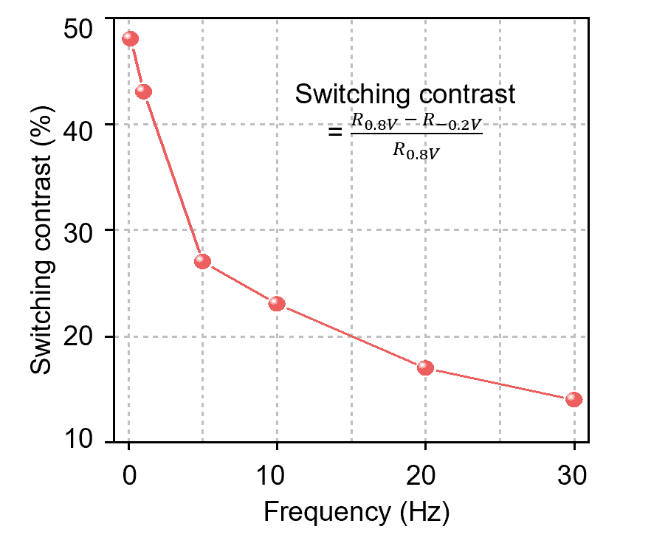
Fig. S27. Switching contrast of the *r*-GT resonator versus the applied frequency.

#
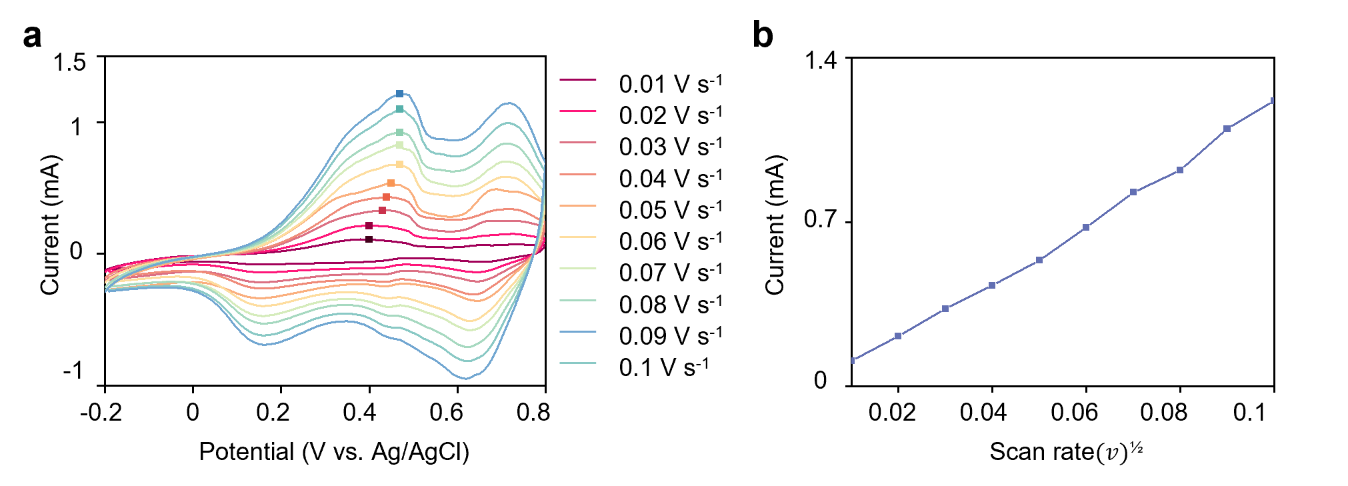
Fig. S28. Cyclic voltammetry of PANI. (a) CV curve of PANI at different scan rates from 0.01 V s^-1^ to 0.1 V s^-1^. (b) The peak current of the first oxidation state increases with dependence on the scan rate by the following equation:

$$\begin{aligned} i_{p}=0.4463 nFAC\left( \frac{nFvD}{RT} \right)^{\frac{1}{2}}\#\left( 12 \right) \end{aligned}$$

Where the $i_{p}$ is the current maximum of the CV curve, n is number of electrons transferred in the redox event, $A$ is electrode area, $F$ is Faraday’s constant, $D$ is diffusion coefficient, $C$is concentration of PANI, $v$ is scan rate, $R$ is Gas constant, and $T$ is temperature. As a result, the $i_{p}$ can be obtained from the slope of a plot against $\sqrt{v}$ at 25°C, using following equation:$\begin{aligned} i_{p}=2.69 \times{10}^{5}n^{\frac{3}{2}}AC\sqrt{Dv} \sim\sqrt{v}\#\left( 13 \right) \end{aligned}$

#
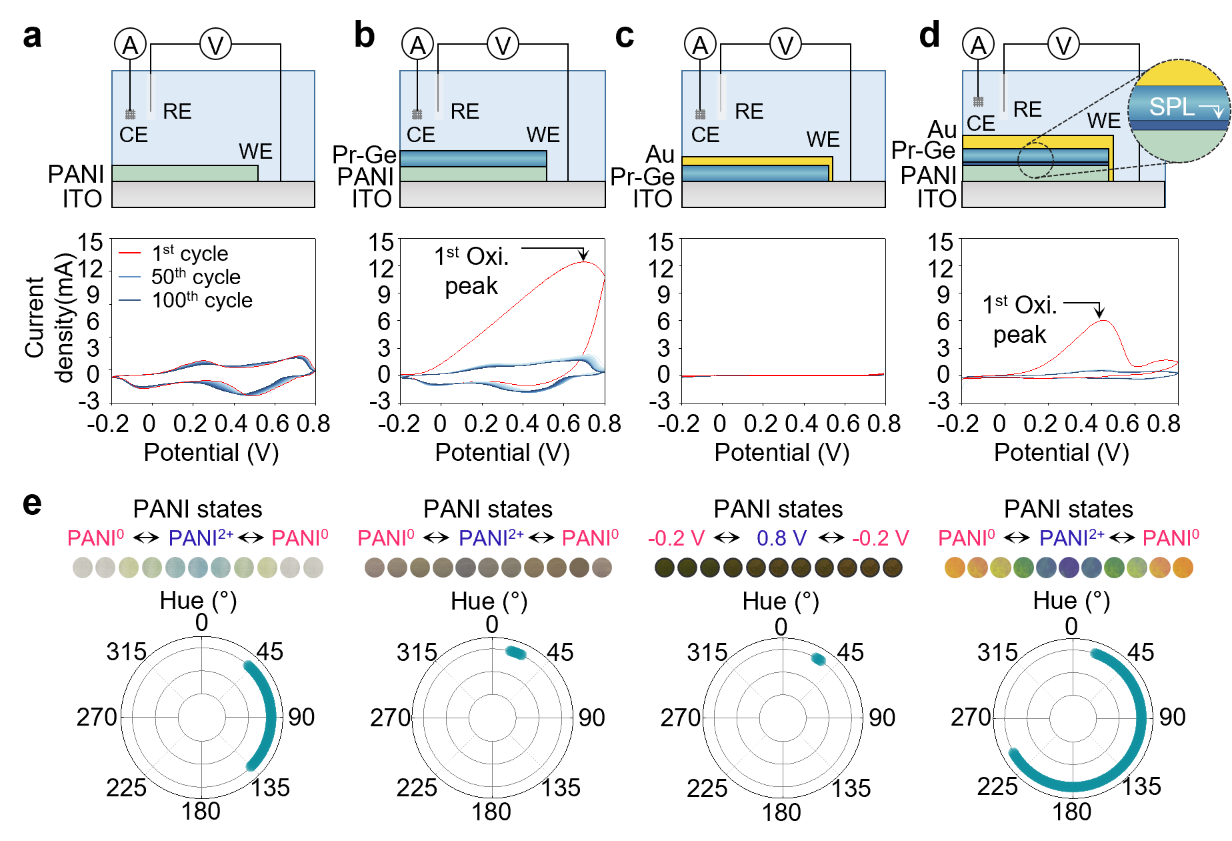
Fig. S29. Self-passivation layer (SPL) and comparison of CV curves with different device structures. Schematics of different device structures, along with the corresponding CV curves: (a) PANI/ITO, (b) Pr-Ge/PANI/ITO, (c), Au/Pr-Ge/ITO and (d) Au/Pr-Ge/PANI/ITO. Cyclic potentiation was conducted using the essential components of Pt mesh(counter electrode, CE), Ag/AgCl electrode (reference electrode, RE), and working electrode (WE). (a) Over 100 cycles, PANI on a conductive substrate exhibits a stable CV curve in the electrolyte without any structural degradation. (b) A peak current of approximately 10 mA was observed during the first cycle, accompanied by only limited colour modulation. After first cycle, the Pr-Ge layer directly exposed to the electrolyte dissolved after the first cycle, resulting in colour modulation similar to that of the PANI/ITO structure*^34^*. (c) Cyclic voltammetry at the Au/Pr-Ge/ITO interface reveals no peak indicative of electrochemical reactions, confirming interfacial inertness. The lack of redox activity precludes SPL formation at this junction, and no associated colour change is observed. (d) Structures incorporating Au which has acts as both a reflector and a protective layer against excessive oxidation in the porous medium. It shows an oxidation peak near 0.4 V during the first cycle but maintains stable cycling afterward. (e) The optical modulation resulting from the applied potential exhibits a limited colour modulation range and low chromaticity due to the absence of a well-defined optical structure. However, the *r*-GT structure, with a self-passivation layer formed through appropriate oxidation, provides reversible and diverse colour display.

#
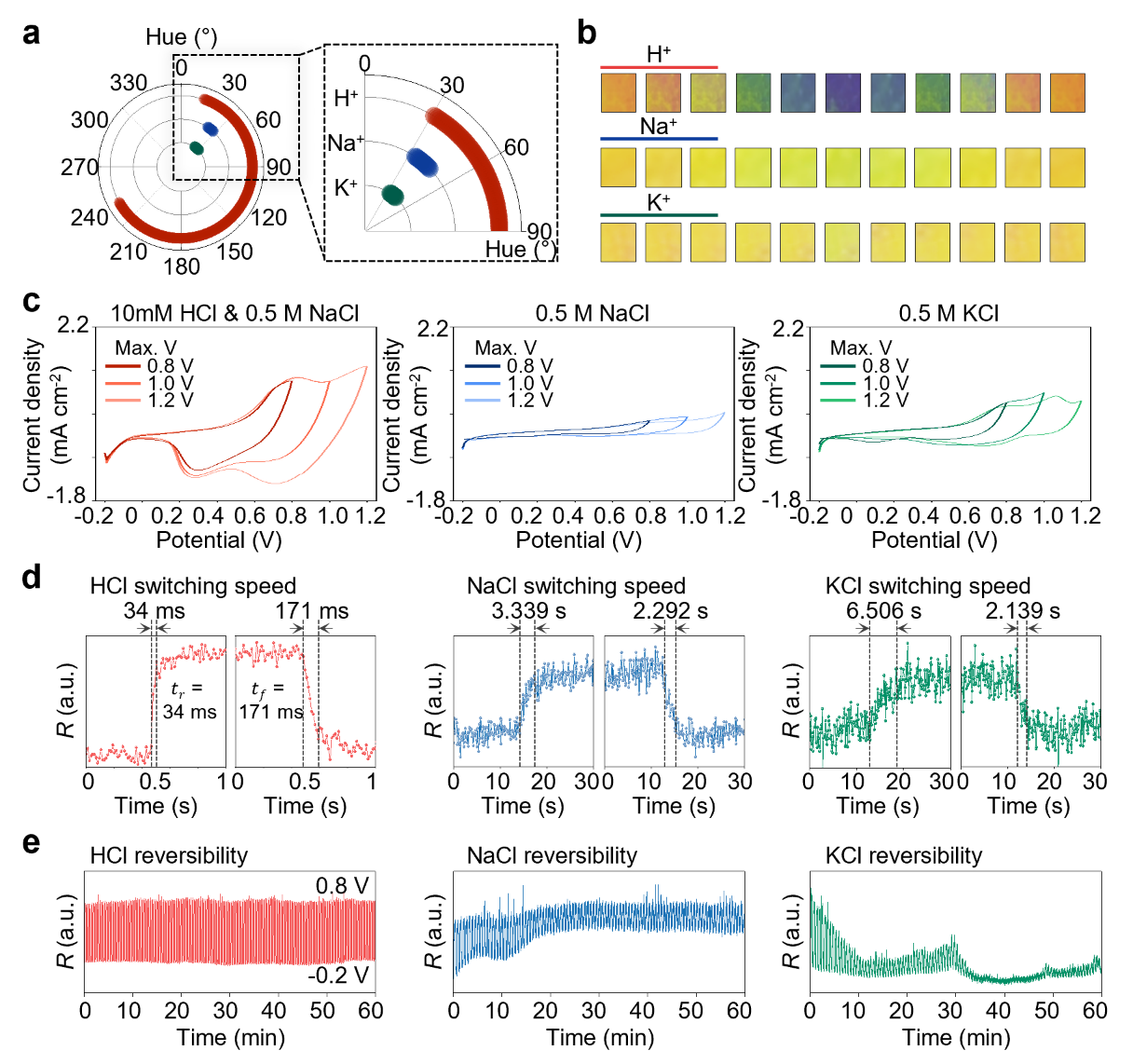
Fig. S30. Comparison of chromatic and electrochemical characteristics associated with different counterions. (a) Variation in hue range and (b) colour variation of *r*-GT in response to counterions H^+^, Na^+^, and K^+^. (c) Evaluation of PANI redox characteristics: This section explores the necessity of a low pH environment (rich in protons) to fully harness the refractive index modulation of PANI. At neutral pH, protons produced via water electrolysis at high potential can sustain redox activity. However, this may cause damage to the polymer chains and reduce reversibility, thereby compromising device stability. Observations suggest that water electrolysis occurs in KCl environments even at low potentials, underscoring the importance of low pH for effective redox reactions and robust chemical passivation to maintain structural stability. (d) Response time and (e) reversibility test of *r*-GT in relation to the counterions H^+^, Na^+^, and K^+^, with only the H^+^ case exhibiting reliable and fast responses.

#
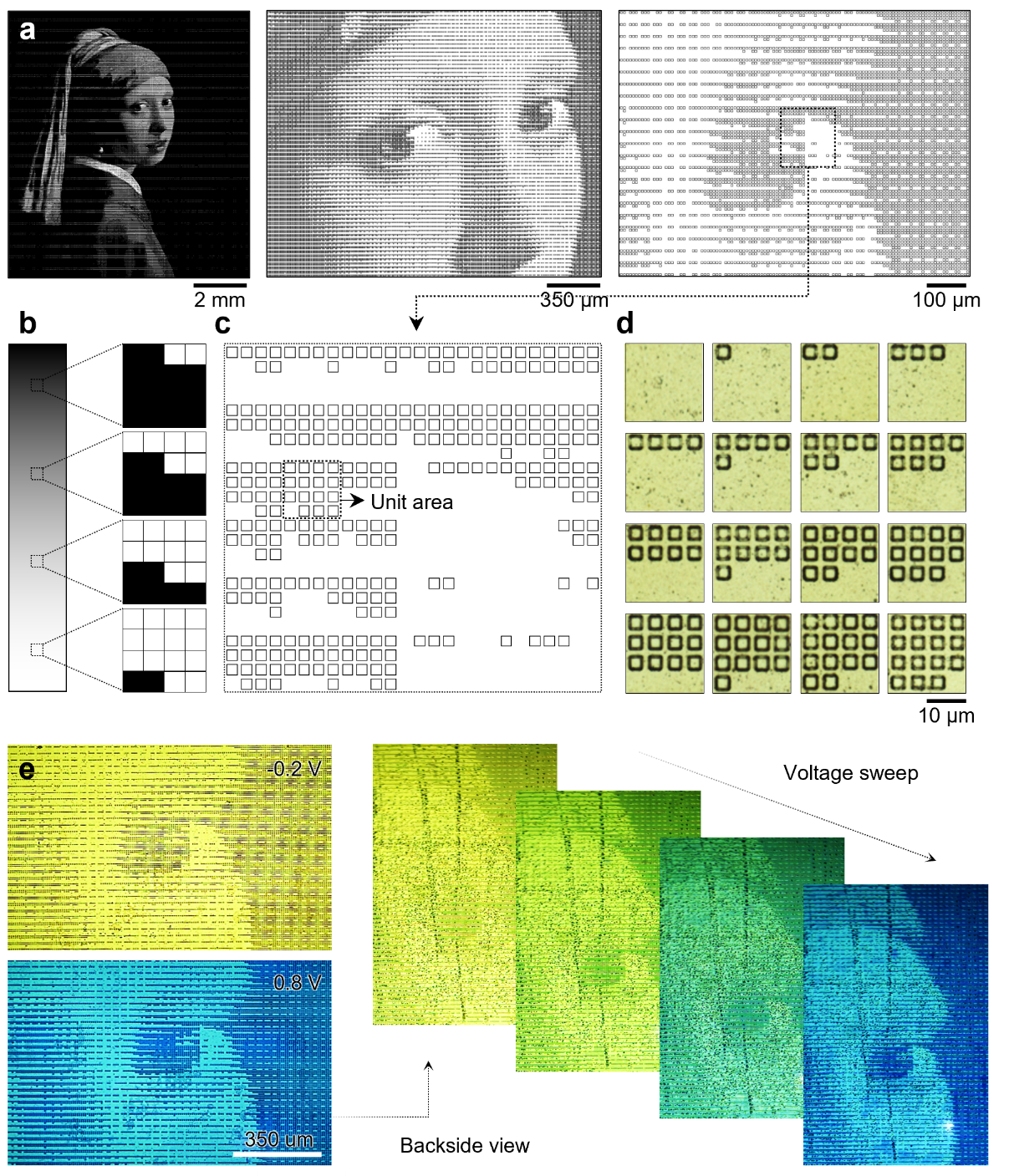
Fig. S31. High-density (HD) *r*-GT monopixel array. (a) Painting pattern of *r*-GT monopixels was inspired by the artwork *"The Girl with a Pearl Earring"* as presented in Figure 4. Scale bars are 2 mm, 350 μm, 100 μm. (b) Schematic illustrating of the expression of grayscale by varying dot density based on Bayer pattern. (c) Magnified view of painting pattern shown in (a), focusing on a unit area. (d) Optical microscopy images of the unit area, demonstrating 4-bit reflection intensity modulation by changing the number of 4 micron-dots. Scale bar is 10 μm. (e) Different side views of HD *r*-GT monopixel arrays under applied voltages at -0.2 V to 0.8 V. Scale bar is 350 μm.

#
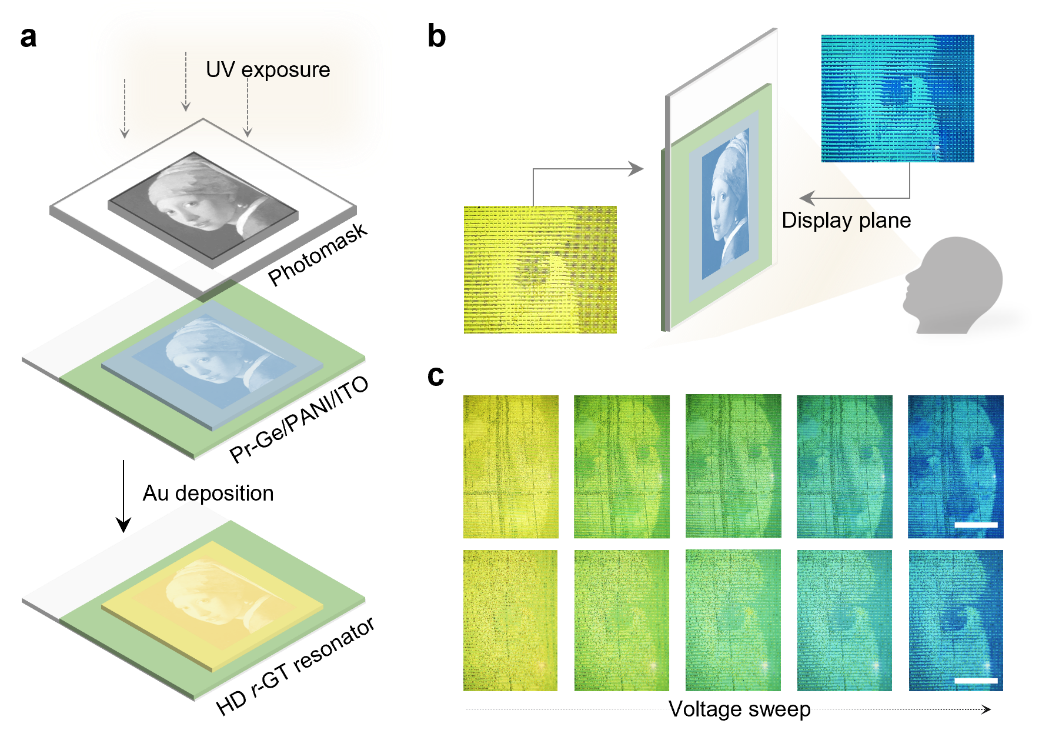
Fig. S32. High-density (HD) patterned *r*-GT resonator. (a) Simplified fabrication process of HD *r*-GT monopixel array with prefabricated painting pattern from “*Girl with a pearl earring*”. (b) Optical microscopy image of high contrast pattern on the display plane. (c) Visible modulation and magnified view of painting pattern with unit area during voltage sweep from -0.2 V to 0.8 V. Scale bar is 500 μm (top) and 250 μm (bottom).

#
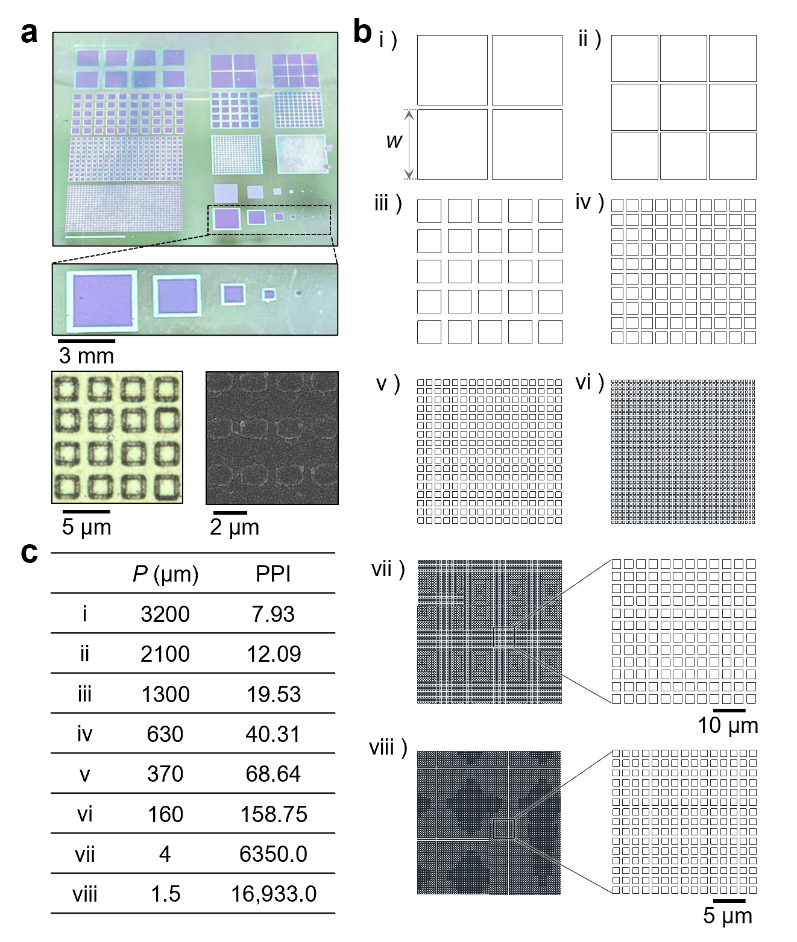
Fig. S33. Design of the large-area, high-density *r*-GT monopixel array. (a) Optical images and scanning electron microscope (SEM) image of fabricated large-area *r*-GT resonator used to calculate pixels per inch (PPI), along with a magnified view of the monopixels. (b) Patterns designed for different pixel pitches (*P*), ranging from 3 mm to 1.5 µm (i-viii). (c) PPI values calculated for each pixel pitch from 3200 μm to 1.5 μm shown in (b).

#
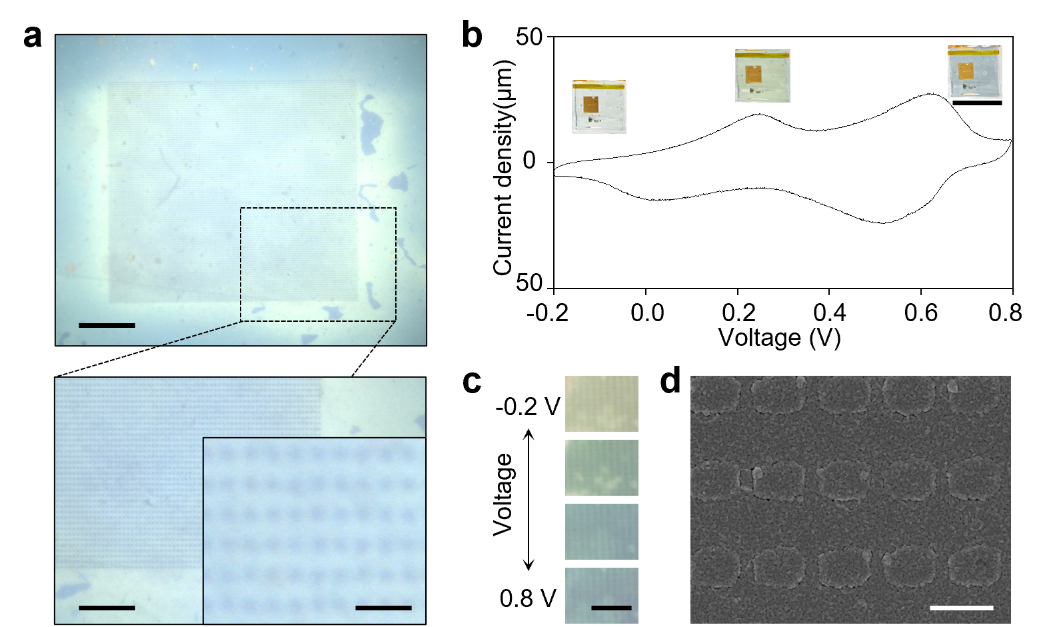
Fig. S34. Electrochemical characterization of micropixel *r*-GT resonator. (a) Optical microscope (OM) images of the *r*-GT micropixel structure at different magnifications. Scale bars are 50 μm, 20 μm, and 5 μm. (b) Cyclic voltammetry curve of the micropixel *r*-GT under electrochemical operation and a photograph of the actual sample. Scale bar is 1 cm. (c) OM images of the micropixel under applied potentials, showing voltage-dependent colour modulation. Scale bar is 10 μm. (d) Scanning electron microscope (SEM) image of the *r*-GT micropixel structure. Scale bar is 1.5 μm.

#
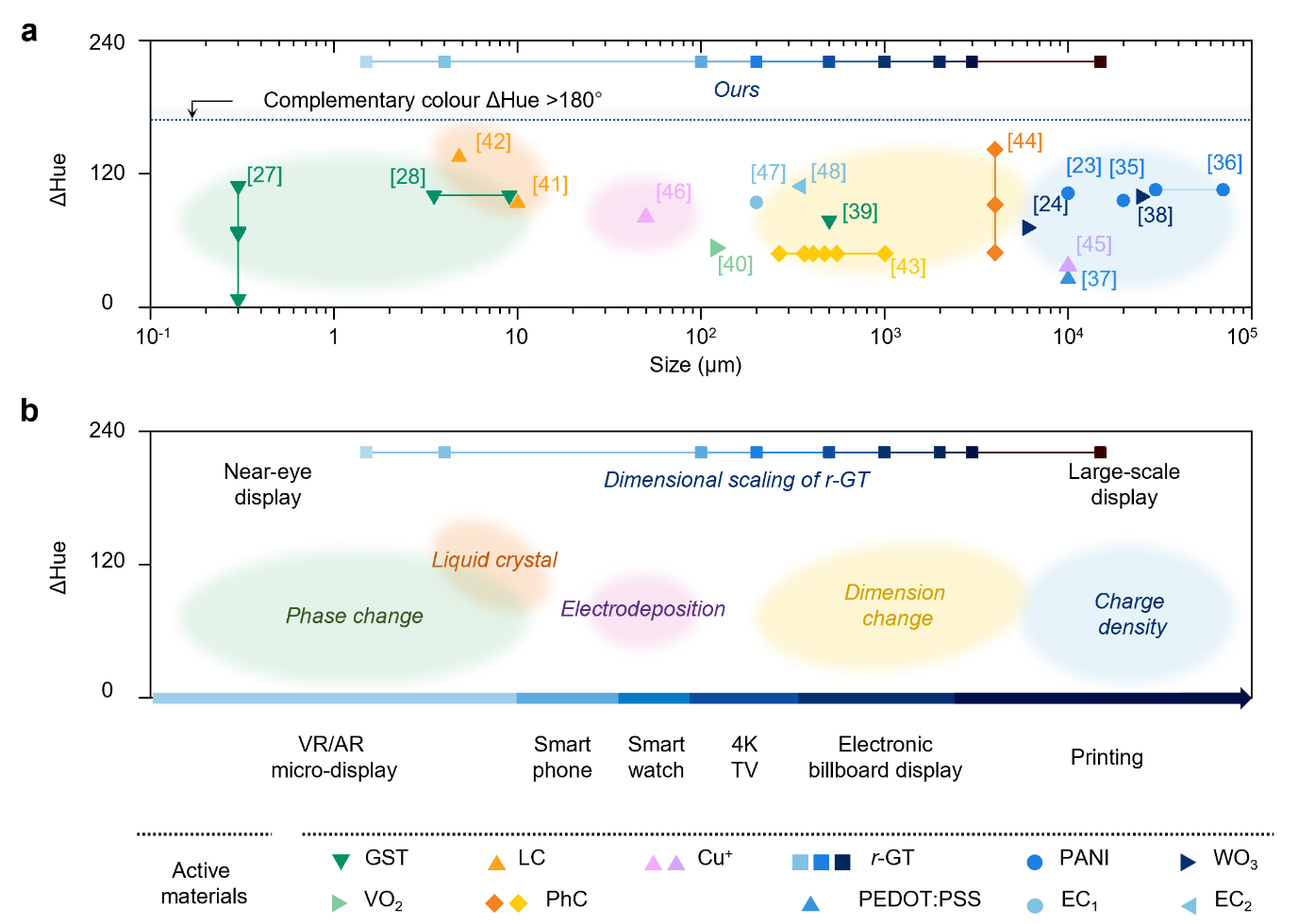
Fig. S35. Comparison of the hue range and pixel size across various active materials (a) and corresponding their modulation mechanisms (b) as shown in Table 6*^23, 24, 27, 28, 35-48^*.

#
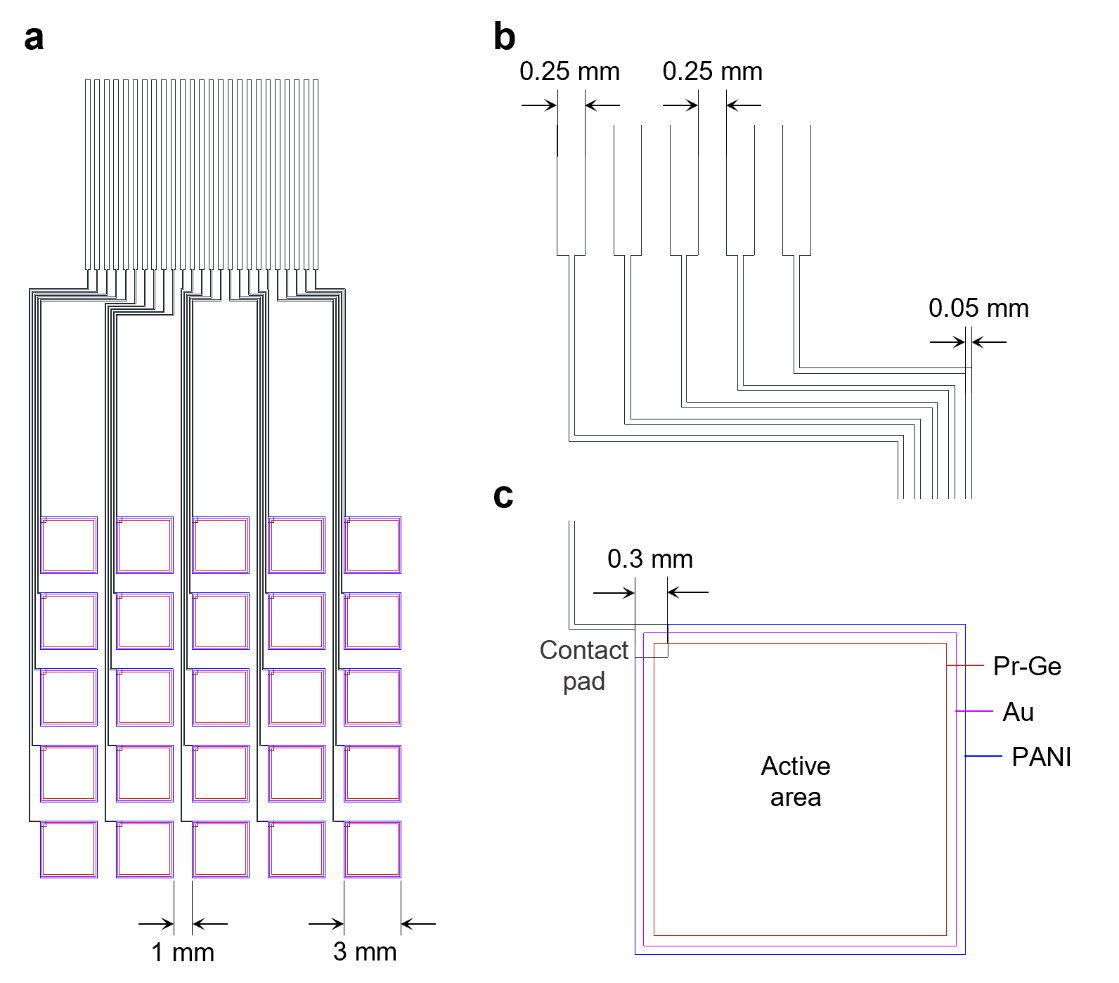
Fig. S36. Mechanical photomask design for the addressable monopixel array design. (a) The design of *r*-GT monopixel array is configured using a 5 × 5 monopixel array. The pixel size and spacing are defined as 3 mm and 1 mm, respectively, by shadow mask. (b) Electrode pattern design of *r*-GT monopixel array for connection with each pixel via photolithography. To enhance pixel density, the electrodes connected to the pixels were designed with a width of 0.05 mm, while the electrode width in the contact region for addressing through the control board was set to 0.25 mm. (c) Active area definition of *r*-GT resonator. The electrode pattern included contact pads between the pixels and electrodes to improve stable electrochemical operation. The PANI region is in direct contact with the electrolyte to facilitate electrochemical operation via protonation. The Au reflector is designed to cover the Pr-Ge region to prevent the direct oxidation of Pr-Ge.

#
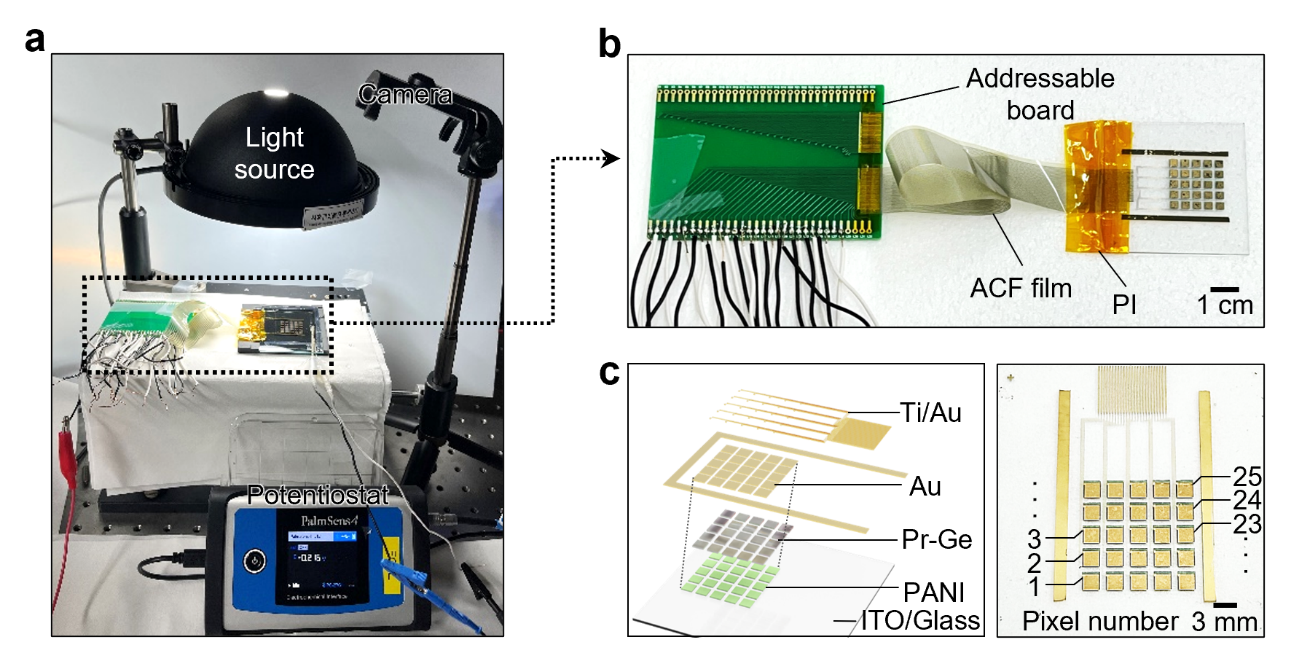
Fig. S37. Operation and measurement setup for addressable function of *r*-GT array. (a) Photograph of the optical measurement setup. Scale bar is 5 cm. (b) Photograph of the individual pixel array with a printed circuit board (PCB) and 5 × 5 monopixel array, where each unit pixel is separately connected to the working electrodes using an anisotropic conductive film (ACF). Scale bar is 1 cm. (c) Schematic showing etch layers of the addressable *r*-GT monopixel array. Each pixel is designated with pixel numbers from “1” to “25” Scale bar is 3 mm.

#
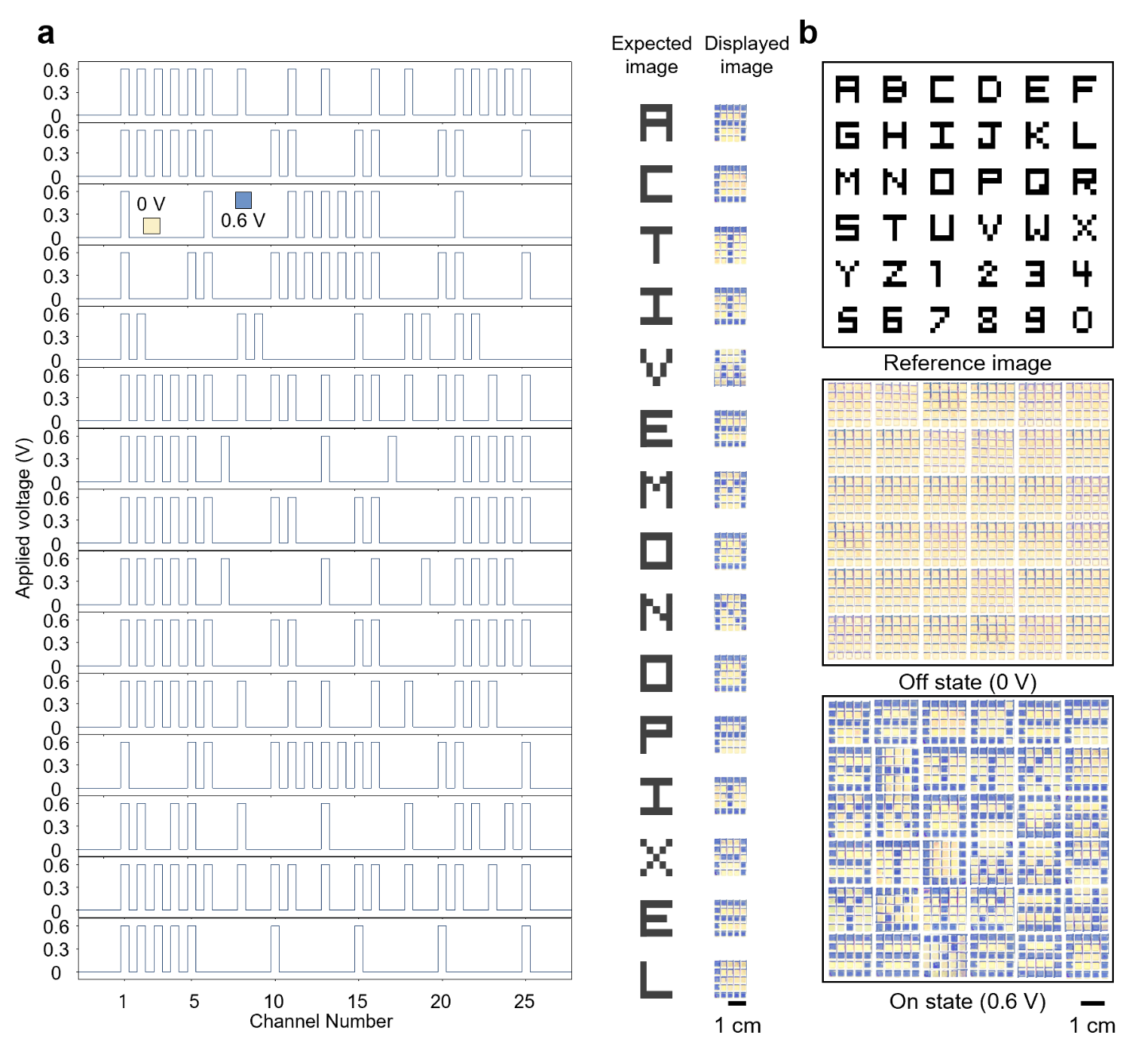
Fig. S38. *r*-GT monopixel array with pixel addressing property. (a) 25-pixelized *r*-GT monopixel array which presents pixel information of “ACTIVE MONOPIXEL” with 25-monopixel array. Scale bar is 1 cm. (b) Reference image of representative pixel information including capital letters from “A” to “Z” and numbers from “0” to “9” (top). Photograph of the addressable *r*-GT monopixel array with 36 individual sequences at 0 V (off state, middle) and 0.6 V (on state, bottom). Scale bar is 1 cm. The metastable electrochemical characteristics of PANI allow for energy-efficient operation by ensuring that the driving voltage is selectively applied only to addressed pixels. Non-addressed pixels remain electrically isolated, thereby effectively preventing unintended activation and minimizing parasitic power consumption.

#
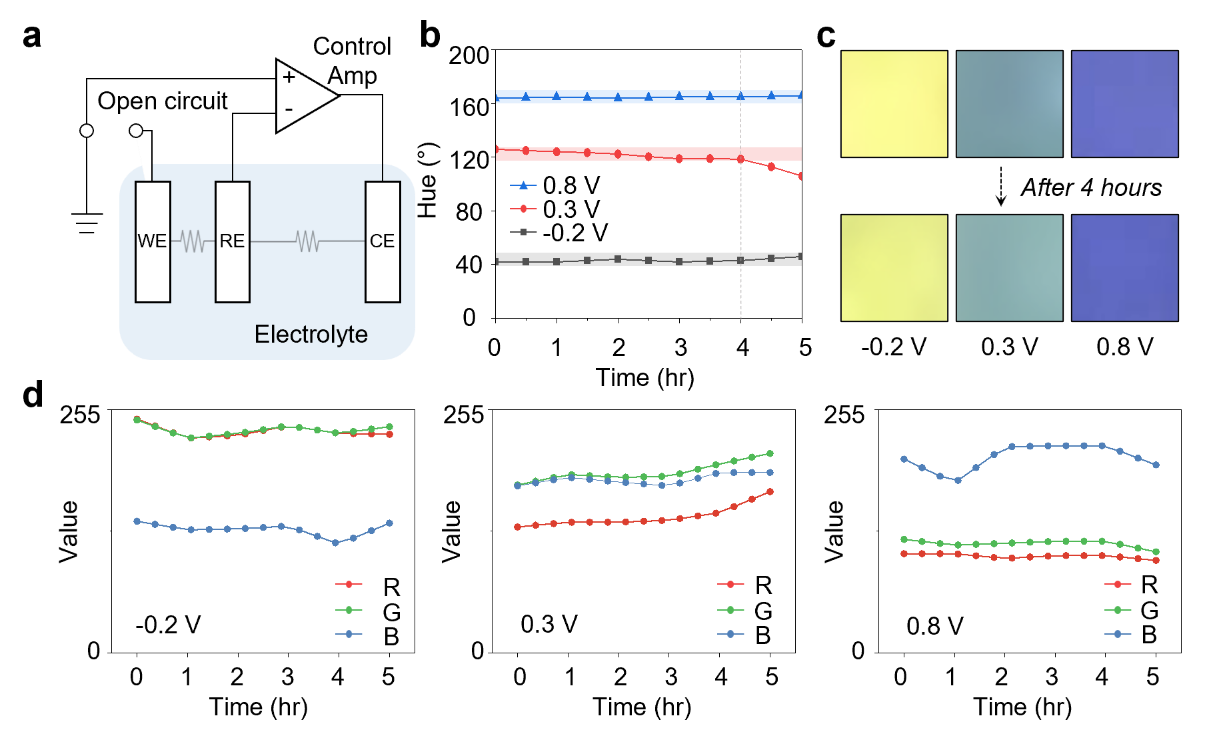
Fig. S39. Metastable properties of *r*-GT monopixel. (a) Simplified potentiostat circuit diagram of three-electrodes system in an open-circuit state. In a three-electrode system, the working electrode (WE), reference electrode (RE), and counter electrode (CE) are connected indirectly through the electrolyte. WE is short-circuited in the circuit after the measurement starts to demonstrate the metastable states. (b) Optical memory effect of the *r*-GT monopixel at different voltages. In electrochemically stable states, such as 0.8 V and -0.2 V, the hue variation remains within 5 degrees over 5 hours. In contrast, at 0.3 V, significant colour degradation becomes apparent after 4 hours. (c) Photographs of *r*-GT resonator after disconnecting the power supply and after 4 hours. (d) RGB values calculated from the optical images at different voltages corresponding to (b).

#
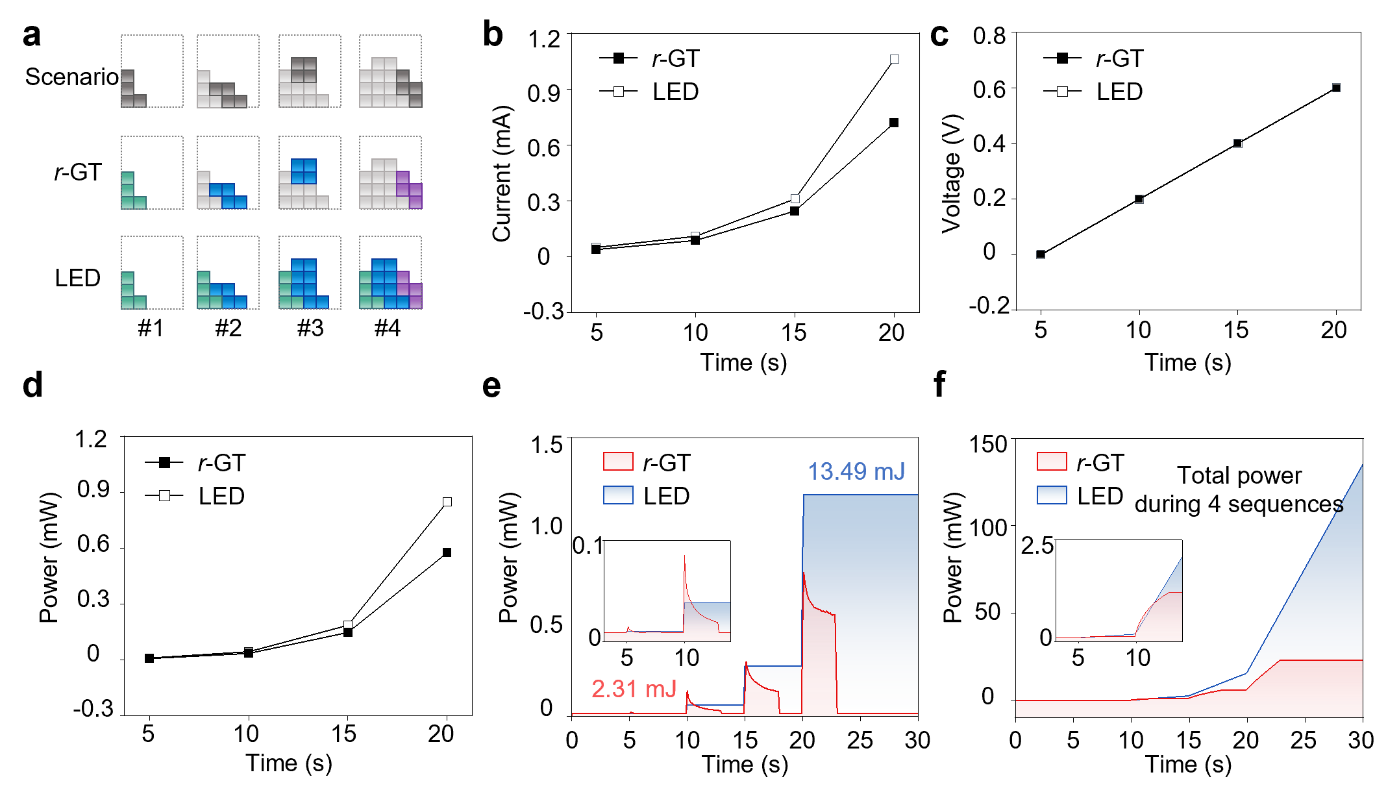
Fig. S40. Non-volatile properties of *r*-GT monopixel array. (a) Schematics depicting a virtual scenario of the *“Tetris game”* and the time-dependent states displayed through *r*-GT and LED. The regions with coloured pixels consume power, while the gray pixels conserve electrical energy. The *r*-GT monopixels, with their optical memory properties, operate only the pixels that are progressively activated based on the scenario, while the volatile LED requires constant power to sustain all pixels. (b and c) The measured current (b) and voltage (c) of the *r*-GT for colour display, compared to the typical voltage and current values of an LED. (d) Calculated power corresponding to different current and voltage values, as shown in (b and c), by time delay. (e) The changed power used to activate the added pixels and the total energy. (f) Calculated total power of *r*-GT and LED for expressing the four-sequences. In comparison to the *r*-GT resonator, LED requires a continuous power supply due to optical volatile properties, resulting in significant power consumption.

#
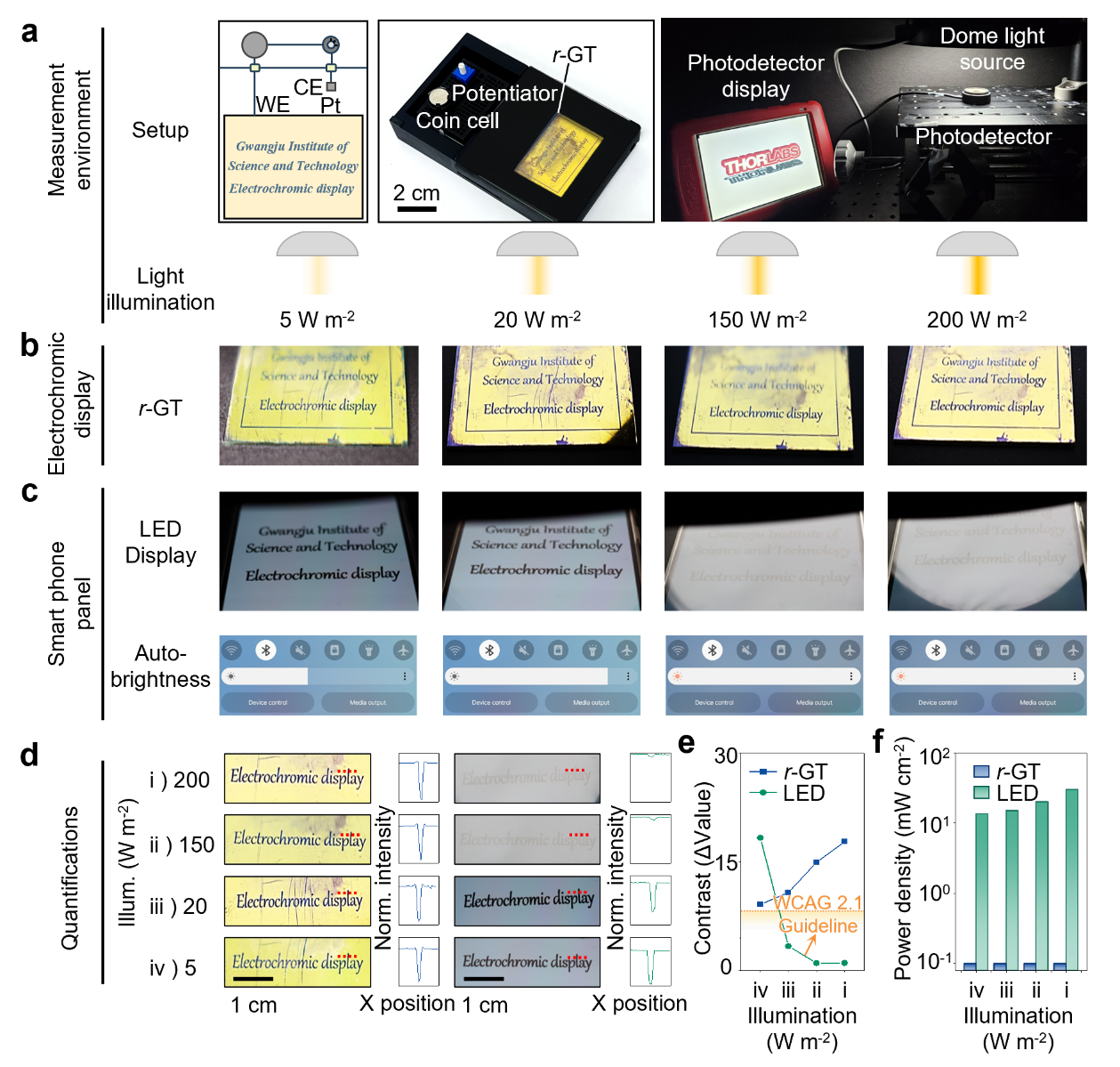
Fig. S41. Device configuration and visibility measurement setup. (a) This panel describes a portable *r*-GT display setup powered by a coin cell and featuring a printed *r*-GT component. Due to its low operational voltage (< 1 V), the coin cell can deliver adequate potential for activating the PANI redox reaction. The measurement setup includes a dome light source and displays for assessing outdoor visibility under strong and varying light conditions (5-200 W m^-2^). (b) Photograph of the *r*-GT under different lighting conditions. (c) Photograph of an LED display (Galaxy S23, Samsung) under various lighting conditions. The images below illustrate the auto-brightness setting. Under intense light, the LED display exhibits poor visibility, even at maximum brightness. (d) Photograph of the *r*-GT resonator and LED. Measured colour contrast of those displays under the different illumination conditions. The dashed red line indicates the pixel region selected for contrast quantification, obtained by grayscale conversion and normalization of intensity. (e) Comparison of visibility between *r*-GT and LED displays under different illumination conditions. Contrast is calculated as the difference between the maximum and minimum intensity (ΔValue) within the red dashed line in (d). For the LED, contrast below the yellow line indicates insufficient visibility for effective visual information delivery. (f) Calculated power density of our device and that of LED which consumes power to adjust to the environmental condition.

#
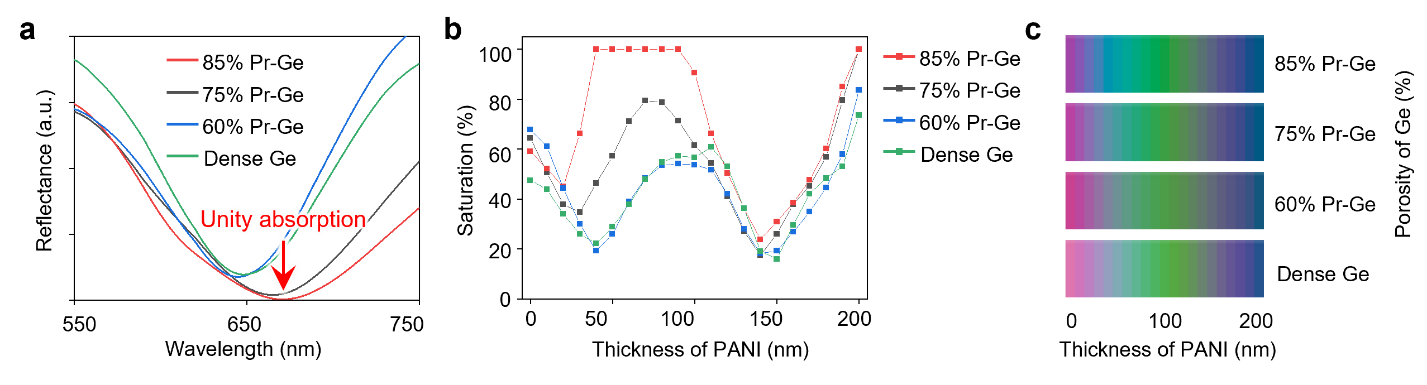
Fig. S42. Optimization of colour modulation and saturation by controlling Ge layer porosity (a) Calculated reflectance spectra of GT resonator as a function of Ge layer porosity (dense, 60%, 75%, and 85%). (b) Comparison of calculated reflected colour saturation with varying Ge porosity. The *r*-GT structure with 85% Pr-Ge achieves near-unity absorption and the highest colour saturation; however, considering fabrication feasibility, we optimised the design using 75% Pr-Ge. (c) Colour chart showing the simulated visible colours as a function of PANI thickness under each porosity condition.

#
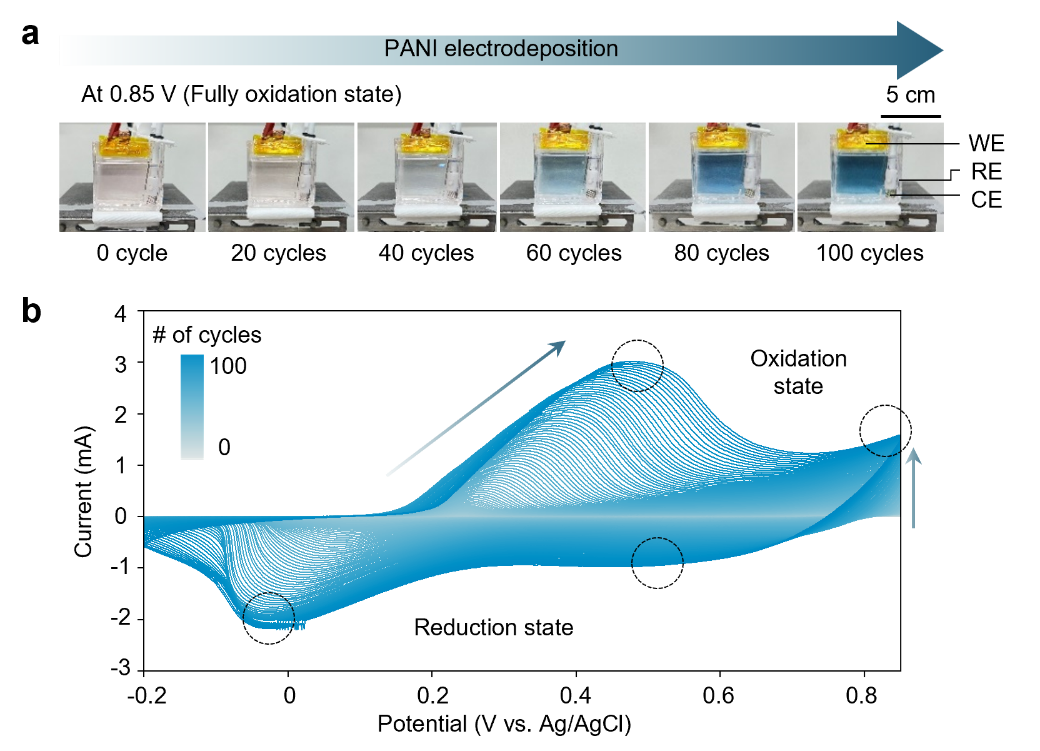
Fig. S43. Electrodeposition process of PANI. (a) Photograph of the electrodeposition process of PANI during 100 cycles in 2 M HNO_3_ and 70 mM aniline monomer. RE, CE, and WE are abbreviations for the reference electrode, counter electrode and working electrode, respectively. The scale bar is 5 cm. (b) The oxidation peaks of the cyclic voltammetric (CV) curve continuously increase due to the deposition of the PANI film.

#
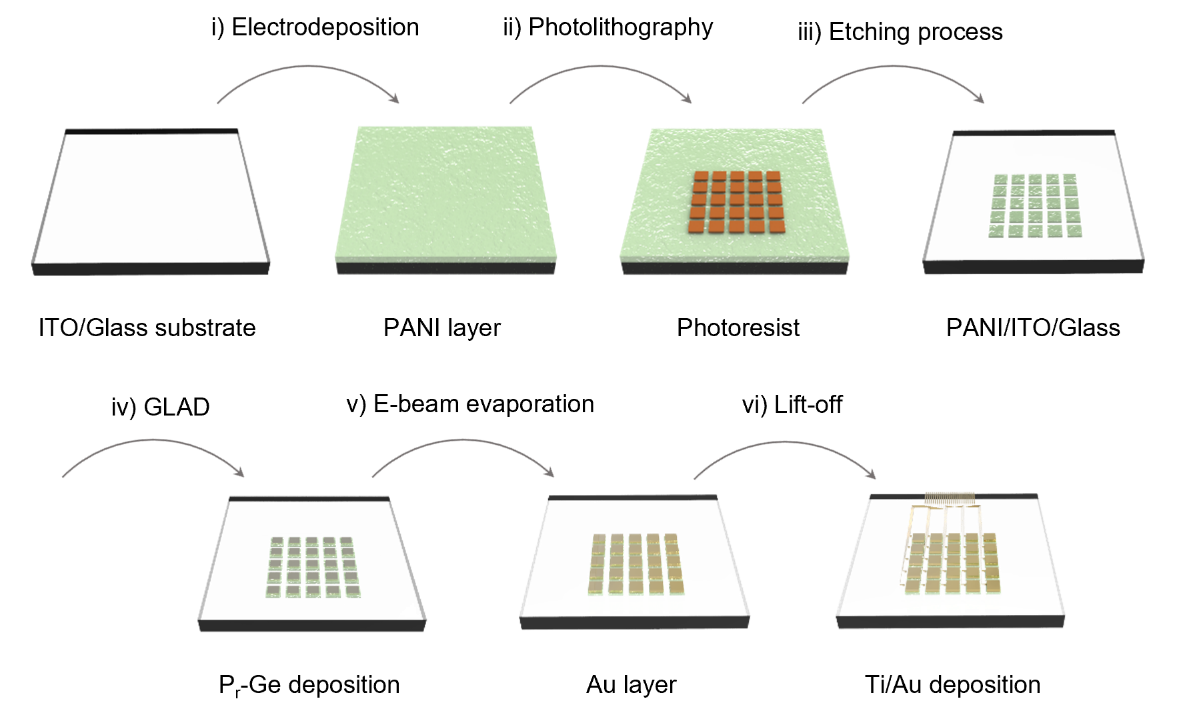
Fig. S44. Schematic illustration of a fabrication step for *r*-GT monopixel array. i) PANI layer is deposited using potentiostat on an ITO/Glass substrate. ii) The photoresist (PR) is patterned using a Cr photomask by photolithography process. iii) To define the isolated pixel area, the PANI layer is etched with O_2_ plasma etching process, and 25 pixels are isolated through the ITO wet etching process. iv) To enhance chromaticity by utilizing the impedance matching process, the porous (Pr) Ge layer is deposited by e-beam evaporation with glancing angle deposition (GLAD) method via a shadow mask. The *r*-GT resonator is configured with an ultrathin and highly lossy film under normal incidence, consisting of an air/absorber/metal mirror configuration. v) The Au layer, serving as the metal reflector, is deposited via e-beam evaporation using a shadow mask. vi) To individually control each pixel, image reversal PR pattern is formed with a Cr mask. Then, the Ti/Au electrodes are deposited with e-beam evaporator and patterned through lift-off process using acetone, where the Ti layer serves as the adhesion layer.

| **Active material** | **Tuning mechanism** | **ΔV (V)** | **ΔHue (∘)** | **Ref** |
| --- | --- | --- | --- | --- |
| PANI | Electrochemical doping | 1.1 | 21.73 | [22] |
|  |  | 2.5 | 102.76 | [23] |
|  |  | 3 | 96.24 | [35] |
|  |  | 5 | 105.78 | [36] |
| PEDOT:PSS | Electrochemical doping | 2.2 | 98.94 | [49] |
|  |  | 6 | 25.81 | [37] |
| WO_3_ | Electrochemical doping | 1.3 | 31.39 | [31] |
|  |  | 1.3 | 110.41 |  |
|  |  | 0.8 | 93.83 | [50] |
|  |  | 0.8 | 76.93 |  |
|  |  | 3 | 71.73 | [24] |
|  |  | 1.2 | 99.62 | [38] |
|  |  | 1.6 | 46.60 | [51] |
| P3MT/PB | Electrochemical doping | 2 | 160.76 | [25] |
| V_2_O_5_ | Electrochemical doping | 2.8 | 56.84 | [52] |
| GST | Phase transition | 5 | 68.14 | [27] |
|  |  | 5 | 7.16 |  |
|  |  | 5 | 109.07 |  |
|  |  | 5 | 66.45 |  |
|  |  | 4 | 100.56 | [28] |
|  |  | 10 | 78.29 | [39] |
| VO_2_ | Phase transition | 2 | 53.42 | [40] |
| AIST | Phase transition | 10 | 2.99 | [53] |
|  |  | 10 | 120.52 |  |
|  |  | 10 | 30.77 |  |
| PhC | Particle rearrangement | 7 | 48.93 | [43] |
|  |  | 3.5 | 49.41 | [44] |
|  |  | 3.5 | 92.41 |  |
|  |  | 3.5 | 141.90 |  |
| **PANI** | **Electrochemical doping** | **1** | **220.67** | **This work** |

Table S1. Comparison of active materials used in tunable photonics with the *r*-GT resonator based on voltage range and hue modulation*^22-25, 27, 28, 31, 35-40, 43, 44, 49-53^*.

| **Active material** | **Mechanism** | **Hue range** | **Volage**  **range** | **Number of states** | **Number of pixels** | **Retention time (hr)** | **Minimum pixel size (𝜇m)** | **ref** |
| --- | --- | --- | --- | --- | --- | --- | --- | --- |
| GST | Phase transition | 78.29 | 10 | 2 | 4 | ∞ | 500 | [39] |
| VO_2_ | Phase transition | 53.42 | 2 | 60 | 144 | 0 | 120 | [40] |
| PhC | Particle rearrangement | 48.39 | 7 | 2 | 7 | - | 1800 | [43] |
| EC | Electrochemical doping | 160.76 | 2 | 3 | 36 | 1 | 5000 | [25] |
| EPD | Electrophoretic particle rearrangement | 38.98 | 15 | 5 | 67200 | ∞ | 176 | [54] |
| **PANI** | **Electrochemical doping** | **220.67** | **1** | **100** | **25** | **15** | **5** | **This work** |

Table S2. Comparison of reported tunable photonics and commercial displays comparing display performance data of six factors*^25, 39, 40, 43, 54^*.

| **Type** | **Active material** | **Structure** | | **ΔHue (∘)** | **ΔV (V)** | **FoM** | **Ref.** |
| --- | --- | --- | --- | --- | --- | --- | --- |
| **CP** | **PANI** | **Planar** | **ITO glass/PANI/**  **Pr-Ge/Au** | **220.6741** | **1** | **220.6741** | **Ours** |
| **CP** | PANI |  | antibody/PANI/  ITO SPE | 132.0991 | 1.9 | 69.5258 | [32] |
| **CP** | PANI |  | CV-GS PANI/ITO/PET | 96.32485 | 1.2 | 80.2707 | [33] |
| **Oxide** | WO_3_ |  | WO_3_/W | 114.7382 | 1.3 | 88.2601 | [31] |
| **CP** | PANI | **Nanostructure** | Nanoparticle | 188.7531 | 1.5 | 125.8354 | [29] |
| **Oxide** | WO_3_ |  | Nanohole | 177.5193 | 3 | 59.1731 | [30] |
| **PCM** | GST |  | Conductive Atomic Force Microscopy | 109.0724 | 5 | 21.8145 | [27] |
| **PCM** | GST |  | Conductive Atomic Force Microscopy | 100.565 | 2 | 50.2824 | [28] |

Table S3. Comparison of optical performance between planar and nanostructured resonators, specifically in terms of hue range and operating voltage (FoM = ΔHue/ΔV)*^27-33^*.

| **Type** | **Active material** | **Structure** | | **ΔHue (∘)** | **ΔV (V)** | **Ref.** |
| --- | --- | --- | --- | --- | --- | --- |
| **CP** | **PANI** | **Planar** | **ITO glass/PANI/Pr-Ge/Au** | **220.6741** | **1** | **Ours** |
| **CP** | T34bT |  | Glass/Cr/Au/T34bT/Al | 177.4432 | 1.7 | [57] |
| **CP** | PANI |  | antibody/PANI/  ITO SPE | 132.0991 | 1.9 | [32] |
| **CP** | PANI |  | CV-GS PANI/ITO/PET | 96.32485 | 1.2 | [33] |
| **CP** | PEDOT:PSS |  | PET/Ag electrode/  electrolyte/CE | On/off | 5 | [61] |
| **Oxide** | WO3 |  | WO3/W | 114.7382 | 1.3 | [31] |
| **PCM** | GST |  | Ag/Sb2S3/Ag/GST | 77.12241 | 10 | [39] |
| **CP** | T34bT | **Nanostructure** | Nanohole | CIE | 1.6 | [57] |
| **CP** | PProDOTMe2 |  | Nanocave | 157.3991 | 1.3 | [55] |
| **CP** | LixSi |  | Metasurface | 89.92093 | 1.49 | [58] |
| **ED** | Ag+ |  | Nanodome | 236.1534 | 1.5 | [56] |
| **LC** | LC |  | Imprinted plasmonic surface | 56.25076 | 50 | [59] |
|  |  |  |  | 126.5371 |  |  |
| **CP** | PANI |  | Nanoparticle | 188.7531 | 1.5 | [29] |
| **Oxide** | WO3 |  | Nanohole | 177.5193 | 3 | [30] |
| **PCM** | GST |  | Conductive Atomic Force Microscopy | 68.14034 | 5 | [27] |
|  |  |  |  | 7.16839 |  |  |
|  |  |  |  | 109.0725 |  |  |
|  |  |  |  | 66.45099 |  |  |
| **PCM** | GST |  | Metasurface | 29.30854 | Current pulse | [60] |
| **PCM** | GST |  | Conductive Atomic Force Microscopy | 100.565 | 2 | [28] |

*CP: conductive polymer

*PCM: Phase change materials

*LC: Liquid Crystal

*ED: electrodeposition

Table S4. Comparison of hue modulation range (ΔHue) and operating voltage range (ΔV) between reported planar and nanostructured resonators*^27-33, 39, 55-61^*.

| **Type of cations** | **ΔHue (∘)** | **Response time (s)** | **Reversibility** |
| --- | --- | --- | --- |
| H^+^ | 220 | 0.034 | 200 |
| Na^+^ | 13 | 17.3 | 5 |
| K^+^ | 8 | 21.3 | 5 |

Table S5. Comparison of optical properties of *r*-GT resonator in different electrolytes based on H^+^, Na^+^, and K^+^.

| **Active material** | **ΔHue (∘)** | **Pixel size (𝜇m)** | **Ref** |
| --- | --- | --- | --- |
| PANI | 102.76 | 10000 | [23] |
|  | 96.24 | 20000 | [35] |
|  | 105.78 | 30000 | [36] |
| PEDOT:PSS | 25.81 | 10000 | [37] |
| WO_3_ | 71.73 | 6000 | [24] |
|  | 99.62 | 25000 | [38] |
| GST | 68.14 | 0.3 | [27] |
|  | 7.16 |  |  |
|  | 109.07 |  |  |
|  | 66.45 |  |  |
|  | 100.56 | 9 | [28] |
|  |  | 3.5 |  |
|  | 78.29 | 500 | [39] |
| VO_2_ | 53.42 | 120 | [40] |
| LC | 93.99479 | 10 | [41] |
|  | 135.0851 | 4.8 | [42] |
| PhC | 48.39 | 1005 | [43] |
|  |  | 549 |  |
|  |  | 471 |  |
|  |  | 407 |  |
|  |  | 366 |  |
|  |  | 266 |  |
|  | 49.41 | 4000 | [44] |
|  | 92.41 |  |  |
|  | 141.90 |  |  |
| EC_1_ | 94.55 | 200 | [47] |
| EC_2_ | 108.88 | 350 | [48] |
| Cu^+^ | 81.02 | 50 | [46] |
|  | 37.30 | 10000 | [45] |
| **PANI** | **220.67** | **1.5** | **This work** |
|  |  | **4** |  |
|  |  | **100** |  |
|  |  | **200** |  |
|  |  | **500** |  |
|  |  | **1000** |  |
|  |  | **2000** |  |
|  |  | **3000** |  |
|  |  | **15000** |  |

Table S6. Comparison of pixel size and hue modulation in reported electrically responsive photonic devices*^23, 24, 27, 28, 35-48^*.

Movie S1. Electrically driven colour modulation of the *r*-GT resonator. *r*-GT resonator shows the colour changes of a reflective display controlled by an applied voltage from -0.2 V to 0.8 V. The colour modulation is based on a reversible electrochemical reaction in conducting polymer. The results demonstrate stable and continuous colour tuning at low operating voltages, highlighting the potential for low-power reflective display applications.

References

1. Inoue, S. et al. High-resolution microencapsulated electrophoretic display (EPD) driven by poly-Si TFTs with four-level grayscale. *IEEE Transactions on Electron Devices* **49**, 1532-1539 (2002).

2. Kodaira, T. et al. A flexible 2.1‐in. active‐matrix electrophoretic display with high resolution and a thickness of 100 μm. *Journal of the Society for Information Display* **16**, 107-111 (2008).

3. Liang, R.-C. et al. Electrophoretic display and novel process for its manufacture. US patent 6,788,449 (2004).

4. Comiskey, B. et al. An electrophoretic ink for all-printed reflective electronic displays. *Nature* **394**, 253–255 (1998).

5. Emori, A. Electrophoretic display substrate, method of inspecting same, and electrophoretic display device. European patent 2,853,940 (2018).

6. Dou, Y. et al. Oil motion control by an extra pinning structure in electro-fluidic display. *Sensors* **18**, 1114 (2018).

7. Hayes, R. A. et al. 52.1: a high brightness colour 160 PPI reflective display technology based on electrowetting. *SID Symposium digest of technical papers* **35**, 1412-1415 (2004).

8. Chen, X. et al. Screen printing insulator coatings for electrofluidic display devices. *Physica Status Solidi A* **212**, 2023-2030 (2015).

9. Feenstra, B. J., Hayes, R. A., Camps, I. G. J. Display device. US patent 10,120,183 (2018).

10. Lim, K.-S. et al. P‐67: Wide Bandwidth Reflective Microshutter Blind Panel for Transparent Organic Light‐Emitting Diode Display. *SID Symposium Digest of Technical Papers* **47**, 1389-1391 (2016).

11. Chan, E. K. et al. Continuous color reflective display fabricated in integrated MEMS-and-TFT-on-glass process. *Journal of Microelectromechanical Systems* **26**, 143-157 (2016).

12. Douglass, M. R. Lifetime estimates and unique failure mechanisms of the digital micromirror device (DMD). *1998 IEEE International Reliability Physics Symposium Proceedings. 36th Annual (Cat. No. 98CH36173)* 9-16 (1998).

13. Hong, J. et al. 54.4 L: Late‐News Paper: Single Mirror Interferometric Display–A New Paradigm for Reflective Display Technologies. *SID Symposium Digest of Technical Papers* **45**, 793-796 (2014).

14. McAvoy, G. J. et al. Digital micro-mirror device. US patent 20,100,149,622 (2010).

15. Sun, Z. et al. 49.5: True Glass‐based Micro‐display with 3µm pixel size using Deformed Helix Ferroelectric Liquid Crystal for VR/AR Displays. *SID Symposium Digest of Technical Papers* **52**, 600-603 (2021).

16. Zhibo, S. Fast ultra-high-ppi display for AR/VR and phase modulation device based on deformed helix ferroelectric liquid crystal. Hong Kong University of Science and Technology, Hong Kong, China (2022).

17. Sun, Z., et al. Fringe field effect free high-resolution display and photonic devices using deformed helix ferroelectric liquid crystal. *Liquid Crystals* **48**, 100-110 (2021).

18. Zhang, Q. et al. Novel Frame Buffer Pixel Circuits and Silicon Backplane Development for Polarization-Independent LCOS. *IEEE Photonics Journal* **16**, 2201109 (2024).

19. Muramoto, Y. et al. Retardation compensating plate to compensate residual retardation of a liquid crystal panel, a compensator, a liquid crystal display device, and a projection image display apparatus having the same. US patent 8,018,557 (2010).

20. Han, W. et al. Active Property–Structure Integrated Reconfiguration of Individual Resonant Nanoparticles. *ACS applied materials & interfaces* **16**, 2836-2846 (2024).

21. Ashraf, M., Chapiro, A. & Mantiuk, R. K. Resolution limit of the eye: how many pixels can we see? *Nature Communications* **16**, 9086 (2024).

22. Peng, J. et al. Scalable electrochromic nanopixels using plasmonics. *Science advances* **5**, eaaw2205 (2019).

23. Park, H. et al. A skin-integrated transparent and stretchable strain sensor with interactive color-changing electrochromic displays. *Nanoscale* **9**, 7631-7640 (2017).

24. Li, Y. et al. Colorful electrochromic displays with high visual quality based on porous metamaterials. *Advanced Materials* **35**, 2300116 (2023).

25. Kim, D. S. et al. Low power stretchable active-matrix red, green, blue (RGB) electrochromic device array of poly (3-methylthiophene)/Prussian blue. *Applied Surface Science* **471**, 300-308 (2019).

26. Rodriguez Fernandez, M., Zalama Casanova, E. & Gonzalez Alonso, I. Review of display technologies focusing on power consumption. *Sustainability* **7**, 10854-10875 (2015).

27. Hosseini, P., Wright, C. D. & Bhaskaran, H. An optoelectronic framework enabled by low-dimensional phase-change films. *Nature* **511**, 206-211 (2014).

28. Yoo, S. et al. Multicolor changeable optical coating by adopting multiple layers of ultrathin phase change material film. *ACS Photonics* **3**, 1265-1270 (2016).

29. Zhang, S. et al. Solution-processable multicolor TiO₂/polyaniline nanocomposite for integrated bifunctional electrochromic energy storage device. *Applied Surface Science* **607**, 155015 (2023).

30. Gugole, M. et al. Electrochromic inorganic nanostructures with high chromaticity and superior brightness. *Nano Letters* **21**, 4343-4350 (2021).

31. Wang, Z. et al. Towards full-colour tunability of inorganic electrochromic devices using ultracompact fabry-perot nanocavities. *Nature communications* **11**, 302 (2020).

32. Ranjbar, S. et al. Smart chip for visual detection of bacteria using the electrochromic properties of polyaniline. *Analytical chemistry* **91**, 14960-14966 (2019).

33. Zhou, K. et al. Polyaniline films with modified nanostructure for bifunctional flexible multicolor electrochromic and supercapacitor applications. *Chemical Engineering Journal* **345**, 290-299 (2018).

34. Feldmann, T., Nosrati, S. & Bélanger, F. Solubility of germanium dioxide in commonly used acids—Effect of acid strength, temperature, and water activity. in Extraction 2018: Proceedings of the First Global Conference on Extractive Metallurgy (Springer, Cham, 2018), pp. 2481–2491.

35. Liu, G. et al. Employing polyaniline/viologen complementarity to enhance coloration and charge dissipation in multicolor electrochromic display with wide modulation range. *Journal of Colloid and Interface Science* **655**, 493-507 (2024).

36. Gong, H. et al. Ultrathin flexible electrochromic devices enabled by highly transparent ion-conducting films. *Journal of Materials Chemistry A* **11**, 8939-8949 (2023).

37. Brooke, R. et al. Electrochromic displays manufactured by a combination of vapor phase polymerization and screen printing. *Advanced Materials Technologies* **7**, 2200054 (2022).

38. Zhang, W., Li, H. & Elezzabi, A. Y. Electrochromic displays having two‐dimensional CIE color space tunability. *Advanced Functional Materials* **32**, 2108341 (2022).

39. Prabhathan, P. et al. Electrically tunable steganographic nano-optical coatings. *Nano Letters* **23**, 5236-5241 (2023).

40. Guo, T. et al. Durable and programmable ultrafast nanophotonic matrix of spectral pixels. *Nature Nanotechnology* **19** 1635-1643 (2024).

41. Franklin, D. et al. Polarization-independent actively tunable colour generation on imprinted plasmonic surfaces. *Nature communications* **6**, 7337 (2015).

42. Huang, M. et al. Dynamically Tunable Structural Colors Enabled by Pixelated Programming of Soft Materials on Thickness. Advanced Optical Materials **11**, 2300573 (2023).

43. Fu, Q. et al. Electrically responsive photonic crystals with bistable states for low-power electrophoretic color displays. *Nature Communications* **13**, 7007 (2022).

44. Fu, Q., Zhu, H. & Ge, J. Electrically tunable liquid photonic crystals with large dielectric contrast and highly saturated structural colors. *Advanced Functional Materials* **28**, 1804628 (2018).

45. Meng Z. et al. Tunable Microwave Absorbing Devices Enabled by Rev ersible Metal Electrodeposition. *ACS Applied Materials & Interfaces* **16**, 11686-11693 (2024).

46. Moon, C. W., Kim, Y. & Hyun, J. K. Active electrochemical high-contrast gratings as on/off switchable and color tunable pixels. *Nature Communications* **13**, 3391 (2022).

47. Olsson, O., Gugole, M., Blake, J.C., Chukharkin, M. & Dahlin, A. Electrochromic active matrix with plasmonic metasurfaces. *RSC Applied Interfaces* **1**, 719 (2024).

48. Xu, L.-J. et al. A metallosupramolecular polymer deposited via inkjet printing for fast-switching pixelated electrochromic devices. *Journal of Materials Chemistry C* **10**, 3353-3359 (2022).

49. Rekha, S. el al. ITO-Free Solution-Processed Flexible Electrochromic Devices Based on PEDOT: PSS as Transparent Conducting Electrode. *ACS Applied Materials & Interfaces* **9**, 19427-19435 (2017).

50. Wu, Q. et al. Electrochromic metamaterials of metal–dielectric stacks for multicolor displays with high color purity. *Nano Letters* **21**, 6891-6897 (2021).

51. Chen, J. et al. Reversible active switching of Fano and Fabry–Pérot resonances by electrochromic operation. *Laser & Photonics Reviews* **16**, 2200303 (2022).

52. Mjejri, I., Rougier, A. & Gaudon, M. Low-cost and facile synthesis of the vanadium oxides V2O3, VO2, and V2O5 and their magnetic, thermochromic and electrochromic properties. *Inorganic chemistry* **56**, 1734-1741 (2017).

53. Ríos, C. et al. Color depth modulation and resolution in Phase-Change Material nano-displays. *Advanced Materials* **28**, 4720-4726 (2016).

54. Zang, H. et al. Electrophoretic display comprising black, white, red, and yellow particles. *Journal of the Society for Information Display* **30**, 387-394 (2022).

55. Xiong, K. et al. Video‐rate switching of high‐reflectivity hybrid cavities spanning all primary colors. *Advanced Materials* **35**, 2302028 (2023)

56. Wang, G. et al. Mechanical chameleon through dynamic real-time plasmonic tuning. *ACS Nano* **10**, 1788-1794 (2016).

57. Rossi, S. et al. Dynamically tuneable reflective structural coloration with electroactive conducting polymer nanocavities. *Advanced Materials* **33**, 2105004 (2021).

58. Yang, L. et al. Rechargeable metasurfaces for dynamic color display based on a compositional and mechanical dual-altered mechanism. *Research* **2022**, 9870321 (2022).

59. Franklin, D. et al. Actively addressed single pixel full-colour plasmonic display. *Nature communications* **8**, 15209 (2017).

60. Wang, Y. et al. Electrical tuning of phase-change antennas and metasurfaces. *Nature Nanotechnology* **16**, 667-672 (2021).

61. Ersman, P. A., Kawahara, J. & Berggren, M. Printed passive matrix addressed electrochromic displays. *Organic electronics* **14**, 3371-3378 (2013).
